# Durability of Flax Fiber Reinforced Polymer Sheets Under Hygrothermal Conditions: Tensile and Shear Performance

**DOI:** 10.3390/ma19183822

**Published:** 2026-09-08

**Authors:** Yangyang Xia, Pengyu Li, Lingli Shen, Jifang Niu, Huiguo Zhao

**Affiliations:** 1 College of Engineering, Xizang University, Lhasa 850000, China; lipengyu20000609@163.com (P.L.); njfxzu@163.com (J.N.);; 2 Plateau Major Infrastructure Smart Construction and Resilience Safety Technology Innovation Center, Lhasa 850000, China

**Keywords:** flax fiber composites, hygrothermal aging, tensile strength, interlaminar shear, DMA, long-term performance

## Abstract

Fiber reinforced polymers (FRPs) have gained widespread application in civil engineering due to their light weight, high strength, and excellent corrosion resistance. Among various fibers, natural fibers have recently attracted increasing attention because of their renewability, low cost, and reduced environmental impact. However, natural fiber reinforced polymer composites (NFRPs) suffer from high moisture absorption and inferior mechanical properties, which are further deteriorated under hygrothermal environments, severely limiting their service conditions. This study focuses on flax fiber reinforced polymer composites (FFRPs) and, through systematic experimental testing, investigates their durability under hygrothermal conditions. Hygrothermal aging was conducted at 90% relative humidity and 40 °C for up to 30 days. Water uptake, mechanical properties, and thermomechanical behavior were evaluated for composites fabricated by two molding processes (autoclave and hand-laying) with varying ply numbers (10, 20, 30, 40, and 50 layers). The optimal ply number for mechanical performance was identified, and the influence of the different resin systems and fiber forms between the two processes on the cross-process comparison, as well as the rationale for the 30-layer laminate and the effect of laminate thickness, were clarified. Microstructural changes were examined via scanning electron microscopy. This work provides systematic experimental evidence and data for assessing the long-term durability of FFRP composites in humid and warm environments, offering valuable guidance for their practical application in civil infrastructure.

## 1. Introduction

Fiber reinforced polymer (FRP) composites have experienced rapid development in civil engineering applications due to their advantages of light weight, high strength, and corrosion resistance. Currently, the most widely used reinforcing fibers in composite materials are inorganic fibers such as carbon, glass, and basalt fibers. With growing environmental concerns and increasing ecological awareness, renewable natural fibers are gaining prominence in composite material research and applications. Natural fiber reinforced polymer (NFRP) composites offer benefits including recyclability, cost-effectiveness, and environmental friendliness, leading to their widespread adoption across various fields. However, their susceptibility to moisture absorption, relatively low mechanical properties, and marked degradation under hygrothermal conditions substantially limit their application scope and environmental adaptability.

Fiber reinforced polymer (FRP) is a new type of composite material, mainly composed of high-performance fibers, and polyester-based or epoxy resins. Artificial synthetic fiber composites have the advantages of high strength, a small coefficient of thermal expansion, and high heat deflection temperature. However, they have relatively low impact strength, a large strength dispersion coefficient, high cost, and are difficult to degrade, which has a marked impact on the environment [1]. After years of development, artificial synthetic fiber composite materials have very mature manufacturing processes and customizable technologies. Compared with synthetic fibers, natural fibers have the advantages of being renewable [2], recyclable [3], low-cost and easily degradable [4,5]. Its density is mostly distributed between 1.3 and 1.6 g/cm^3^ [6], which is much lower than that of glass fiber (about 2.5 g/cm^3^) [7].

Natural fiber reinforced composites are widely available, including wood, flax, cotton, hemp, animal hair, etc. Among them, flax fibers are increasingly commonly used as reinforcing composites due to their advantages such as high breaking strength, low elongation, good bending performance, and large torsional stiffness [8,9]. Table 1 [10] shows the densities and mechanical properties of common natural fibers and collectively referred fibers.

The influence of temperature on the mechanical properties of composite materials has always been a concern. Lu Zhongyu [11] conducted a study on the effect of high temperature on basalt fiber reinforced composites. The research found that with the increase in temperature, both tensile and shear properties decreased. Tu, J [12] et al. pointed out that with the increase in temperature, the degradation rate of GFRP (glass fiber reinforced polymer) bars shows marked differences; Starkova O [13] et al. studied the diffusion of water molecules in carbon, glass, and aramid fiber reinforced composites and found that the diffusion coefficients of different fiber composites vary greatly, and the interlayer shear strength of fiberglass reinforced plastic bars decreases by 10–50% in a humid environment. Hong Jijian [14] et al. found that the flexural strength of lightweight composite materials decreased by 65.1% and the tensile strength decreased by 64.4% due to water absorption. To date, research has been conducted on the durability of fiber reinforced composites exposed to high humidity [15] environments and water immersion [16].

The hydrolysis of fibers upon contact with water and the interfacial debonding between fibers and matrix materials are regarded as the main reasons for the decline in mechanical properties. Juan L et al.’s [17,18] research indicates that high temperature and hydrolysis reactions can lead to marked degradation of lignin on the fiber surface. Moudood A [19] et al. studied the hydrothermal aging mechanism of flax fiber-reinforced composites and found that the water absorption behavior of the studied composites concurred with Fickian law. Jia Yunlong et al. [20] pointed out that the mechanical properties of plant fiber reinforced composites would decline in a humid and hot environment, which is directly related to their water absorption behavior.

Some scholars believe that the water absorption of NFRP will cause the interface between the fibers and the matrix to break, thus reducing the tensile strength of the composite material. Current research by some scholars has shown that both the tensile modulus and flexural modulus of NFRP composites will decrease when they come into contact with water and absorb moisture [21]. Qiu Hongjun [22] et al. pointed out in their research that the interference-linked structure of aviation composite materials would cause a decline in their mechanical properties in a humid and hot environment. Zhang Ousheng [23] et al. conducted research on pultruded carbon fiber and glass fiber reinforced composites in distilled water at different temperatures and found that high temperatures would accelerate the debonding between the resin and fiber. In the long term, the shear strength of FFRP decreased to varying degrees. Rajaram A N [24] et al. reported the fact that the tensile, compressive and bending properties of carbon fiber reinforced composites under the effect of a wet heat environment are negatively correlated with water absorption. By constructing a model and comparing the test results, they derived the relationship between the strength distribution parameters of wet heat-aged materials and the absorbed moisture content, etc.

Considerable research efforts have been devoted to understanding the hygrothermal aging behavior of flax fiber composites. Experimental investigations have examined the effects of aging temperature, humidity level, and exposure duration on moisture absorption characteristics and mechanical property degradation [25,26]. The moisture absorption behavior of FFRPs generally follows Fick’s law, with elevated temperatures accelerating the diffusion process. Interestingly, some studies have reported that in the early stage of hygrothermal aging, post-curing of the matrix may lead to a temporary increase in tensile and bending strengths before subsequent degradation occurs [27]. The fatigue behavior of FFRPs under hygrothermal conditions has also been investigated, revealing that combined elevated temperature and moisture content lead to reduced stiffness and strength, with fatigue life decreasing by approximately three orders of magnitude in shear-dominated laminates [28]. Hybridization with synthetic fibers such as glass or basalt has been explored as a strategy to improve durability, and the influence of matrix type on aging resistance has been examined [29].

Furthermore, while extensive research has addressed the hygrothermal aging of FFRPs, systematic studies investigating the combined effects of ply number, fabrication method, and aging duration on the durability of woven flax fabric composites are still scarce. The relationship between composite architecture and hygrothermal degradation mechanisms requires further elucidation. Additionally, further investigation is still required to establish the long-term performance of FFRPs in humid and warm environments and to facilitate their safe and confident use in structural applications.

Recent studies have begun to relate the manufacturing route of flax/polymer laminates directly to their microstructure, processing window and durability. For autoclave-cured thermoset laminates, Stejskal et al. [30] cured unidirectional flax/epoxy prepreg at 120 °C under 3 bar for 1 h and reported markedly lower porosity and scatter than for non-autoclave consolidation, while Schulte et al. [31] applied a −0.9 bar vacuum together with 7 bar autoclave pressure at a 2 °C/min ramp and a 120 °C dwell of 60–90 min, showing that the moisture uptake of the resulting flax/epoxy laminates remained strongly dependent on the consolidation parameters. For thermoplastic matrices, Parodo et al. [32] showed that in flax/polypropylene laminates the timing of vacuum application and the cooling rate govern the fiber–matrix interface and hence the laminate quality. For liquid-molding routes, Martinez and Nutt [33] produced woven flax/vitrimer-epoxy laminates by resin transfer molding (RTM) and obtained flexural strength and a tensile modulus equivalent to those of an aerospace-grade epoxy reference, demonstrating that flax/polymer laminates can be manufactured by closed-mold liquid molding without sacrificing performance. More recently, Parodo et al. [34] compared autoclave thermoforming with hot-press molding for woven flax/PP prepregs and showed that autoclave thermoforming provides more precise pressure and temperature control, and accordingly better consolidation and process repeatability, than direct hot pressing. Together, these works confirm that the consolidation route and its governing parameters (vacuum level, pressure, heating ramp, dwell time and cooling rate) are decisive for the quality and hygrothermal durability of flax/polymer laminates, but they rarely vary the ply number systematically, which is the aspect addressed below.

The present study aims to address these research gaps by comprehensively investigating the durability of flax fiber reinforced polymer composites under hygrothermal conditions through experimental testing. Specifically, flax fabric composites with varying ply numbers (10, 20, 30, 40, and 50 layers) were fabricated using two different molding processes (autoclave and hand-laying) and subjected to accelerated aging at 40 °C and 90% relative humidity for up to 30 days. Water absorption behavior, mechanical properties, and thermomechanical performance were characterized at different aging intervals. The findings of this work are expected to provide experimental insights for the design and application of sustainable flax fiber composites in civil infrastructure.

The scientific purpose and novelty of this work are as follows. Existing reports on the hygrothermal aging of natural fiber composites mostly focus on a single fabrication process, a single ply number, or a single aging duration, and systematic experimental data on the combined effects of ply number, fabrication method, and aging duration are still scarce. By systematically measuring, under the same hygrothermal condition (40 °C, 90% RH, up to 30 days), the water uptake, tensile and interlaminar shear, and dynamic thermomechanical properties of flax fabric composites with different ply numbers (10–50 layers) produced by two molding processes (autoclave and hand-laying), the present study provides a complete set of experimental data not previously presented in the literature, and evaluates the influence of each factor on durability, thereby clarifying the actual scientific contribution of this work.

## 2. Materials and Methods

### 2.1. Test Materials

#### 2.1.1. Linen Fiber Cloth

**Figure 1:**
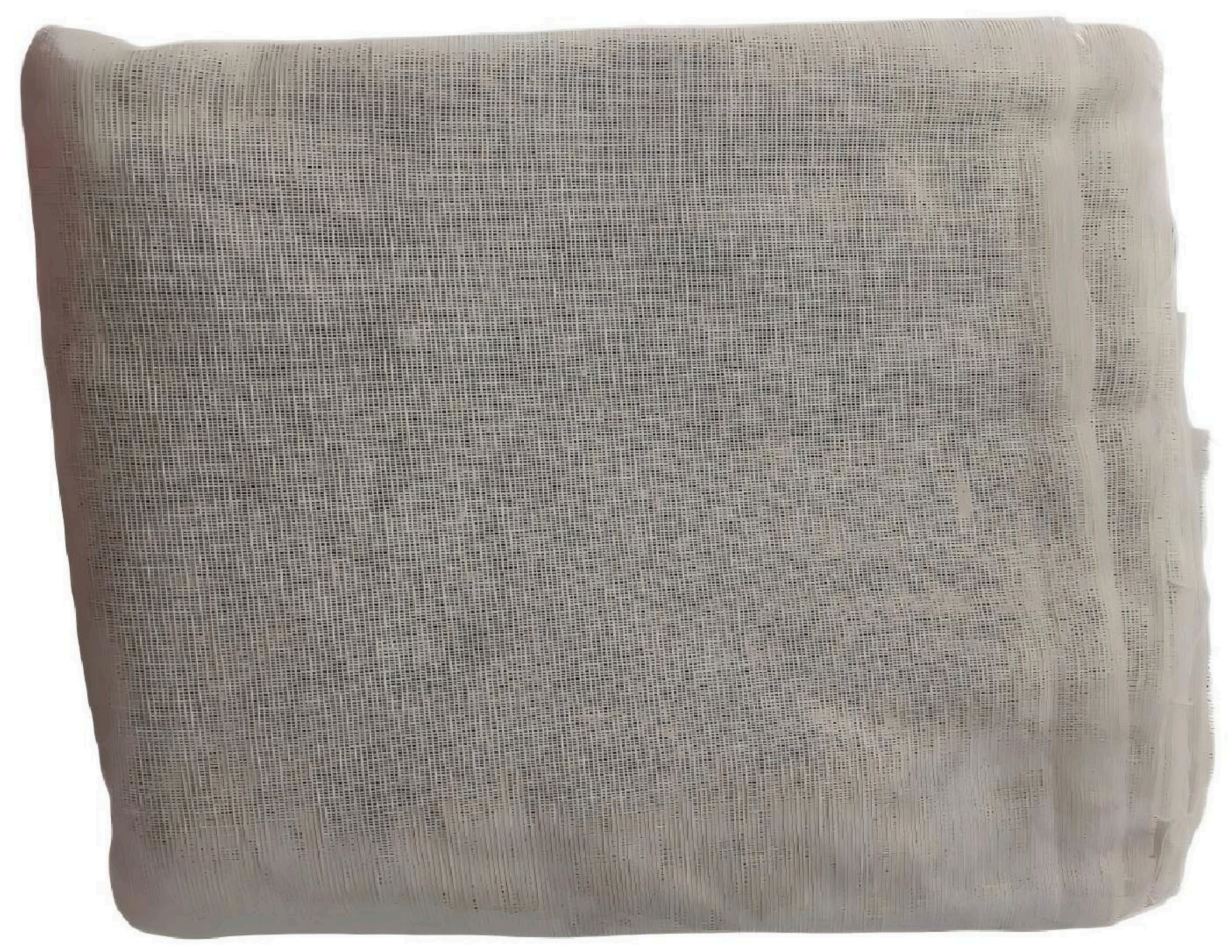
The Linen fiber used for testing.

This paper selects the bidirectional (plain-weave) flax fiber cloth composite with more stable mechanical properties as the reinforcement. The bidirectional flax fiber cloth is provided by Changli Flax Factory, Harbin, China; the fabric areal density is 233 ± 20 g/m^2^ (corresponding to the autoclave prepreg, see Table 2), the fiber areal density is 140 ± 10 g/m^2^, the fiber bulk density is 1.5 g/cm^3^, and the nominal thickness is 0.16 mm. The fabric has a bidirectional orthogonal plain-weave structure in which the warp and weft yarn bundles cross regularly, with uniform yarn spacing and a smooth, non-entangled surface, thereby ensuring the stability of fiber orientation during hygrothermal aging and loading. It is shown in Figure 1.

#### 2.1.2. Base Material

The performance of the matrix material directly affects the performance of the composite material. Since the matrix material is directly exposed to the environment, if it is sensitive to the environment, it will fail prematurely in the actual process, which in turn directly leads to the premature failure of the composite material. The base material of this article is epoxy resin produced by Fyfe Company LLC, San Diego, CA, USA, and its properties are shown in the table below.

### 2.2. Sample Preparation

#### 2.2.1. Preparation of NFRP Sheets by the Hand-Laying Method

**Figure 2:**
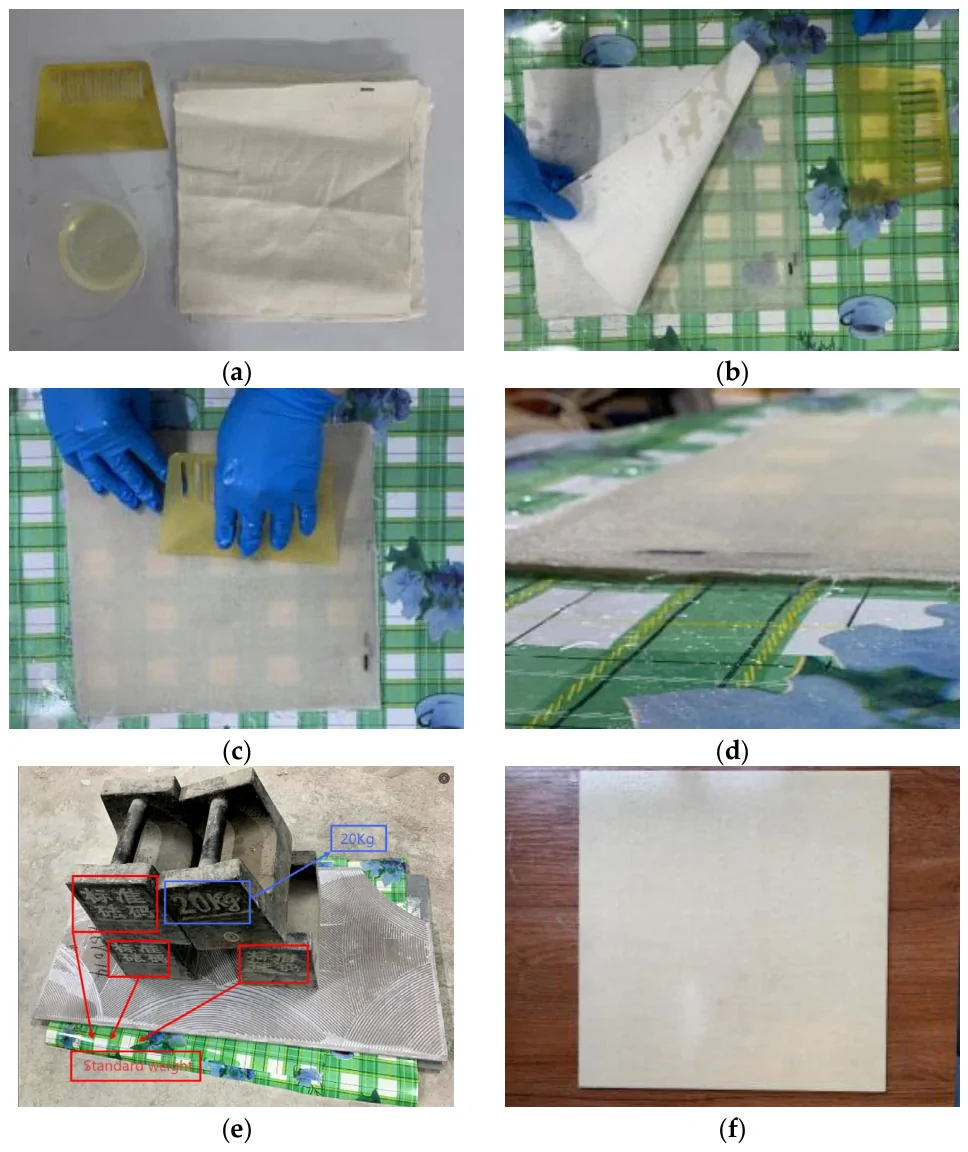
Flowchart for making the hand-laid board. (**a**) Material preparation. (**b**) Laying process. (**c**) Bubble removal. (**d**) Laying completion. (**e**) Pressure-assisted forming. (**f**) Hand-laid FFRP plate.

This article uses the hand-laying process to produce 10-layer hand-laid boards, with flax fiber cloth as the fiber material. The base adhesive is TS-impregnated adhesive, with a tensile strength of 65.65 MPa and a tensile modulus of 3.19 GPa. The amount of adhesive applied to the board is 250 g/m^2^, and the pressure condition is set at 0.1 MPa. The specific production process is shown in Figure 2.

(1) Fiber cloth preparation: Use scissors to cut the flax fiber cloth into 300 × 300 mm^2^, ensuring that the cutting is neat and there are no obvious burrs on the edges. Make marks in the weft direction and stack them neatly to avoid obvious creases on the fiber cloth, which may affect the force on the fibers.
(2) Resin material preparation: Pour the impregnating adhesive solution A and solution B (curing agent) into a disposable plastic bowl in a mass ratio of 1:0.345. Use a pipette to ensure that the error is within the allowable range. It is strictly forbidden to add the curing agent in excess and then draw it out. Use a glass stirring rod to stir the mixed solution in one direction to ensure that the A and B agents are thoroughly and evenly mixed. Stir 400–800 mL for approximately 15 min until there are no obvious bubbles on the surface.
(3) Fiber cloth laying: Use a disposable cotton soft towel dipped in an appropriate amount of alcohol to clean the smooth and flat surface of the floor tiles thoroughly. Lay a layer of PVC film and fix its four corners with adhesive tape to prevent curling and shifting. Pour an appropriate amount of the well-mixed resin evenly onto the plastic film. Lay the pre-cut flax fiber cloth in the center of the floor tiles. Use a plastic scraper to scrape off the excess resin glue and air bubbles on the flax fiber cloth. Then pour an appropriate amount of resin glue and repeat the previous steps until all 10 layers of flax fiber cloth are laid. During the laying process, the linen fiber cloth is arranged layer by layer in the weft direction and with the impregnating adhesive. The sizing amount for flax fiberboard is 250 g/m^2^, and the sizing amount for each layer is uniform. During the laying process, ensure that the excess impregnating glue in each layer is scraped off with a scraper to prevent the formation of air bubbles inside the hand-laid board later, which may affect its mechanical properties.
(4) After all the flax fiber cloth is laid, immediately cover the surface with the same PVC plastic film. Then, use a plastic scraper to scrape off the excess resin glue and air bubbles, and finally cover it with another layer of floor tiles.
(5) Place weights on the upper floor tiles to apply pressure to the flax fiber board with a pressure strength of 0.1 MPa, ensuring that the fiber volume fraction of the produced board is within the set range, there are no holes inside, and the outer surface is flat.

#### 2.2.2. Autoclave Formed FFRP Sheet

In this paper, 10, 20, 30, 40 and 50 layers of FFRP plates were fabricated respectively by the autoclave molding method. The natural fiber selected was RP140- linen fabric, and the resin used was AGMP3600-biomass resin (ACC (Beijing) Science and Technology Co., Ltd., Beijing, China) based on rosin anhydride. The parameters and properties of the linen fabric are shown in Table 3.

During the hot-press forming process, it is necessary to ensure that the pressure on the test piece is not less than −0.08 MPa. The autoclave molding is linearly heated to 90 °C from 0 to 45 min, with a heating rate of 1–3 °C/min. From 45 to 75 min, it is preheated at a constant temperature of 90 °C. After the preheating is completed, it is rapidly heated to 130 °C. The pressure condition should meet 0.5–0.6 MPa. Under this constant temperature and pressure condition, maintain for 120 min, and then immediately cool down until it cools down.

### 2.3. Test Method

It should be emphasized that the two molding processes used different resin systems and different fiber forms. Therefore, the performance differences between them involve confounding variables and cannot be attributed solely to the manufacturing process (see Table 4); all cross-process comparisons in this paper are treated as relative comparisons, and the influence of the resin/fiber systems is acknowledged in the interpretation of the results.

The humidity environment for the durability performance test is formulated in accordance with the ASTM D5229/D5229M-20 specification [35], with a temperature of 40 °C and a relative humidity of 90%. This accelerated aging condition was selected because it can markedly accelerate moisture diffusion and interfacial degradation, while the temperature is not so high as to induce resin thermal degradation that would mask the water-induced degradation mechanism; it thus allows distinguishable durability differences to be obtained within a feasible time (up to 30 days). The temperature and humidity environmental simulation test in this paper adopts the constant temperature and humidity chamber produced by Dongguan Haiheng Testing Instrument and Equipment Co., Ltd., (Dongguan, China) model HIH-HH-1000. To ensure the accuracy of the test environment, a thermometer and hygrometer of model JB913F produced by Medeshi Company (Guangdong Medeshi Instrument & Meter Co., Ltd., Guangzhou, China) was used.

**Figure 3:**
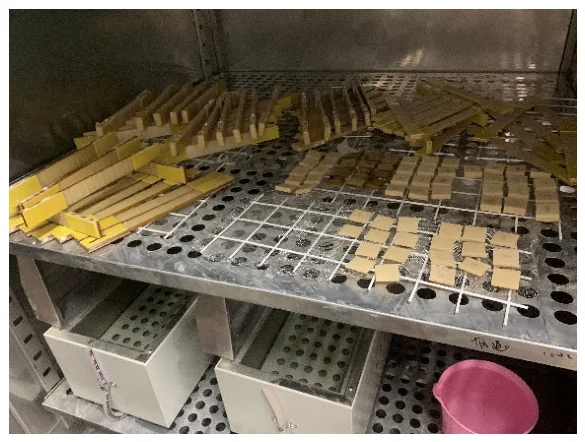
Constant temperature and humidity test chamber.

After the sample is prepared, it should be placed in a constant temperature and humidity chamber with the set temperature and humidity. Observe at regular intervals whether the test environment is stable. The test environment is shown in Figure 3:

#### 2.3.1. Water Absorption Test

Cut the purchased machine-pressed and hand-laid flax fiber composite material boards into appropriate sizes as required. Apply impregnating glue to the exposed sides of the fibers caused by cutting to prevent moisture from entering the fibers too quickly under the effect of damp heat, which may affect the test data.

The water absorption test was conducted in accordance with the ASTM D570 [36] standard and the weighing method was adopted. The test times were respectively set as 12 h, 24 h, 3 days, 15 days and 30 days. Instead of using a constant-mass immersion endpoint, sampling at these fixed time points was adopted because the hygroscopic process of flax fiber composites clearly involves a Fickian diffusion stage followed by a relaxation (non-Fickian) stage, and sampling at fixed time points allows the full sorption kinetics to be described. After taking out the specimen, it was first placed in a sealed bag to ensure that the test was completed as soon as possible within 30 min. After the test was completed, the specimen was immediately returned to the conditioning chamber. In the water absorption analysis, based on Fick’s law of diffusion and introducing a material relaxation coefficient, the diffusion coefficient D and the water absorption rate *M*(*t*) are described as follows, with the symbols defined in Equations (1)–(3).

The specimens of water absorption performance in this paper were fitted by Fick’s law, and it was assumed that the diffusion coefficient *D* was constant. As the aging time increases, water molecules enter the interior of the material, causing the internal structure of the fibers and resins to become loose, the molecular chains between molecules to be disrupted, and de-bonding to occur at the interface. At this point, the water absorption rate of the composite material drops sharply, and the water absorption of the material changes with the destruction of the internal structure. In response to this situation, the relaxation coefficient (*K*) of the material is introduced. The formula for the water absorption rate is as follows:

(1) $$M(t)=M_{m}\left(1+k\sqrt{t}\right)\left\{1-exp\left(-7.3{\left(\frac{D_{t}}{h^{2}}\right)}^{0.75}\right)\right\}$$

In the formula:

*D*—diffusion coefficient;

*h*—specimen thickness (mm);

t—water absorption time (s);

*K*—material relaxation coefficient;

*M_m_*—the water absorption rate of the sample in the saturated water absorption state, obtained by fitting the water absorption curve;

*M*(*t*)—the water absorption rate of the material over time. The calculation formula is as follows:

(2) $$M(t)=\frac{M_{1}-M_{0}}{M_{0}}\times 100$$

In the formula:

*M*_1_—mass of the specimen at a certain moment (g);

*M*_0_—original mass of the specimen (g).

The calculation of the diffusion coefficient adopts the following formula:

(3) $$D=\pi {\left(\frac{h}{4M_{m}}\right)}^{2}{\left(\frac{M_{2}-M_{1}}{\sqrt[{}]{t_{2}}-\sqrt[{}]{t_{1}}}\right)}^{2}$$

where *h* is the specimen thickness (mm), *M_m_* is the moisture content at saturation (%) obtained from Equation (1), *M*_1_ and *M*_2_ are the moisture contents (%) at times *t*_1_ and *t*_2_ (s), respectively, and *D* is evaluated from the initial linear portion of the *M*(*t*) versus *√t* curve, in accordance with ASTM D5229/D5229M-20 [35].

(1) Specimen preparation

**Figure 4:**
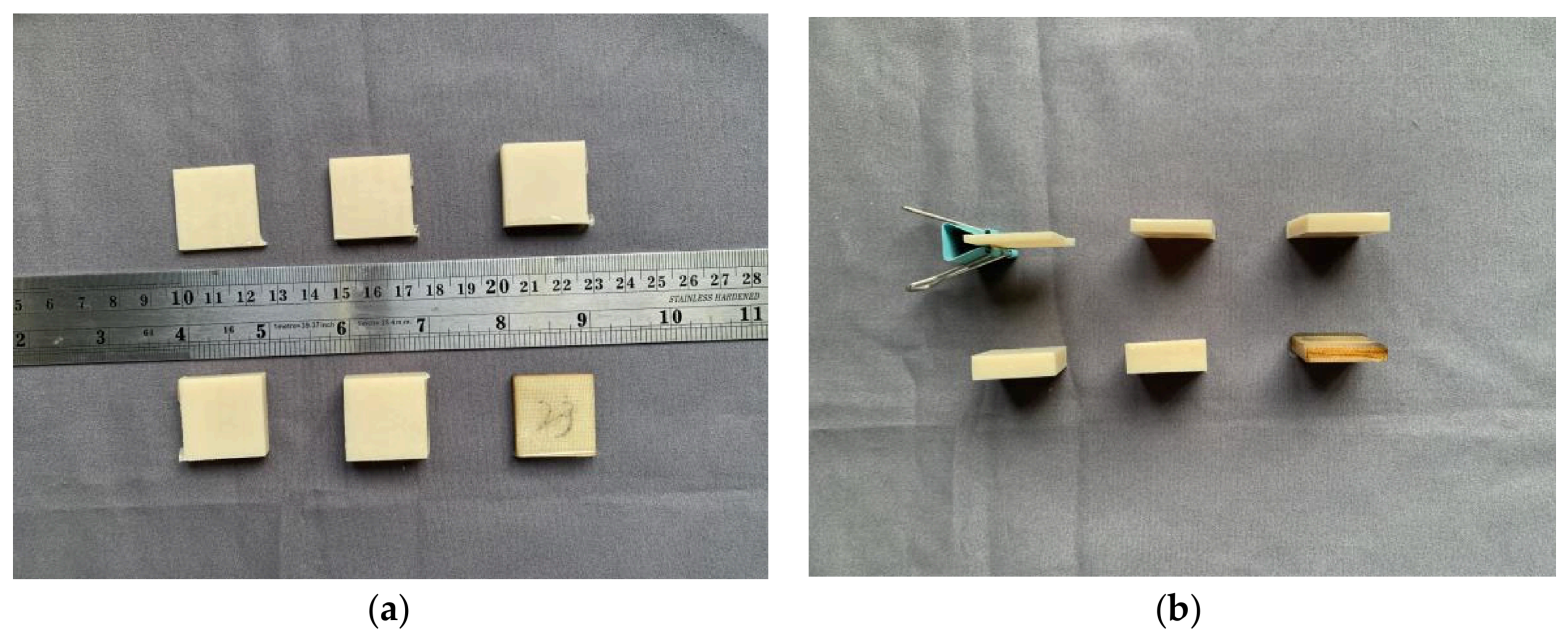
Water absorption specimens with 10–50 autoclave layers and 10 hand-laid layers. (**a**) Front view of water-immersed specimens. (**b**) Side view of water absorption specimens.

Cut the specimen to a size of 250 × 250 mm, and the remaining steps are the same as the interlaminar shear test. The specimens are shown in Figure 4. The first row, from left to right, consists of specimens with 10, 20 and 30 hot-pressed layers. The second row, from left to right, includes specimens with 40 and 50 hot-pressed layers and 10 hand-pressed layers. The surface of the specimens formed by the autoclave molding process is light yellow, while those formed by the hand-laying molding process are yellow.

(2) Specimen design

The water absorption test was divided into 6 groups, with a total of 67 specimens. Among them, 5 groups were formed by the autoclave molding process, namely NFRP with 10, 20, 30, 40, and 50 layers of fibers, totaling 55 specimens. Another group was the control group, which was made of NFRP by hand-laid molding, with a fiber thickness of 10 layers and 12 specimens. The test variables are the different numbers of fiber layers and different aging times. The naming method for specimens is the same as that for tensile tests.

(3) Test contents

The water absorption rate and diffusion coefficient of NFRP under different fiber layers and different aging times were obtained, thereby obtaining the changes in the water absorption performance of NFRP.

#### 2.3.2. Tensile Test

In this paper, the cutting dimensions of the FFRP sheet used in the tensile test by the hand-laying process and autoclave molding process are L × W × T = 250 × 25 × t mm, where t represents the thickness of the sheet. The test method adopts ASTM D3039/D3039M [37], which is commonly used for natural fiber composites. The original gauge length is 50 mm and the tensile rate is 2 mm/min.

(1) Specimen preparation

Due to the relatively low strength of flax fiber board, to prevent the experimental instruments and fixtures from damaging the ends before the test is completed, the cut tensile test board should be ground by an angle grinder at a 45° extension direction within 50 mm on both sides, and then the surface dust should be cleaned thoroughly with a brush. Use a 100-mesh gauze with a ±45° glass fiber sheet, and then clean the surface of the glass fiber with acetone. At the grinding area, use structural adhesive to attach the ground glass fiber sheets (50 × 25 × t) to both ends and fix them with clips. After they have fully cured, remove the clips.

(2) Specimen design

**Figure 5:**
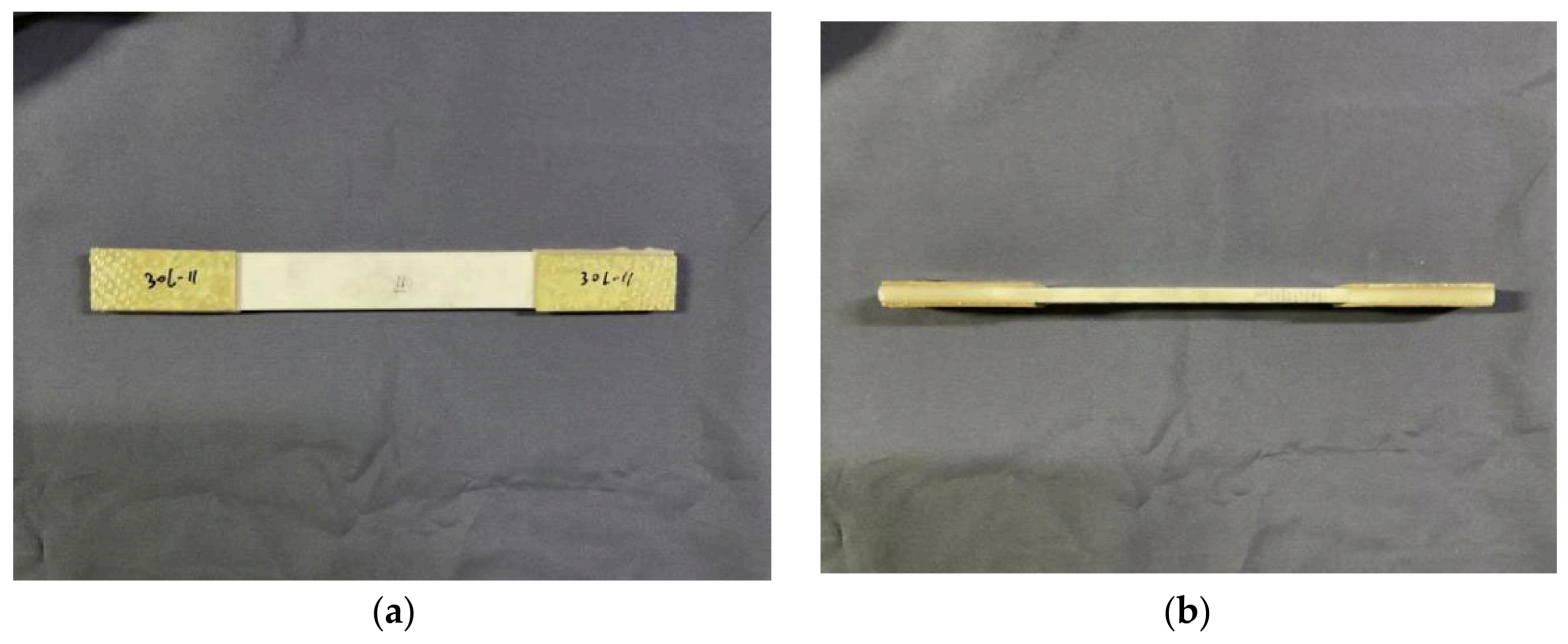
Tensile specimen. (**a**) Front view of tensile specimen. (**b**) Side view of tensile specimen.

This article studies the durability of flax fibers from the perspective of a humid and hot environment. According to its service environment, the test environment in this paper is set as follows: temperature 40 °C, relative humidity 90%. The tensile specimens designed in this experiment are shown in Figure 5. Three independent specimens were tested for each combination of ply number and aging period, i.e., 60 autoclave-molded and 12 hand-laid specimens.

The test was set up in 8 groups, with a total of 72 specimens. The variables of the tensile test were the difference in the number of fiber layers, and the difference in the duration of action. The hand-laid board was used as the control group to observe the degradation of its tensile properties. The main research focuses on the degradation of the tensile strength of fibers with different numbers of layers in a wet and hot environment and fibers with the same number of layers over time. The naming convention for specimens is: NFRP–manufacturing process–number of sheet layers–specimen number. For example, “NFRP-RY-10-1” indicates that the forming process of NFRP is autoclave molding, the number of fiber layers is 10, and the specimen number is 1.

(3) Test apparatus and loading

**Figure 6:**
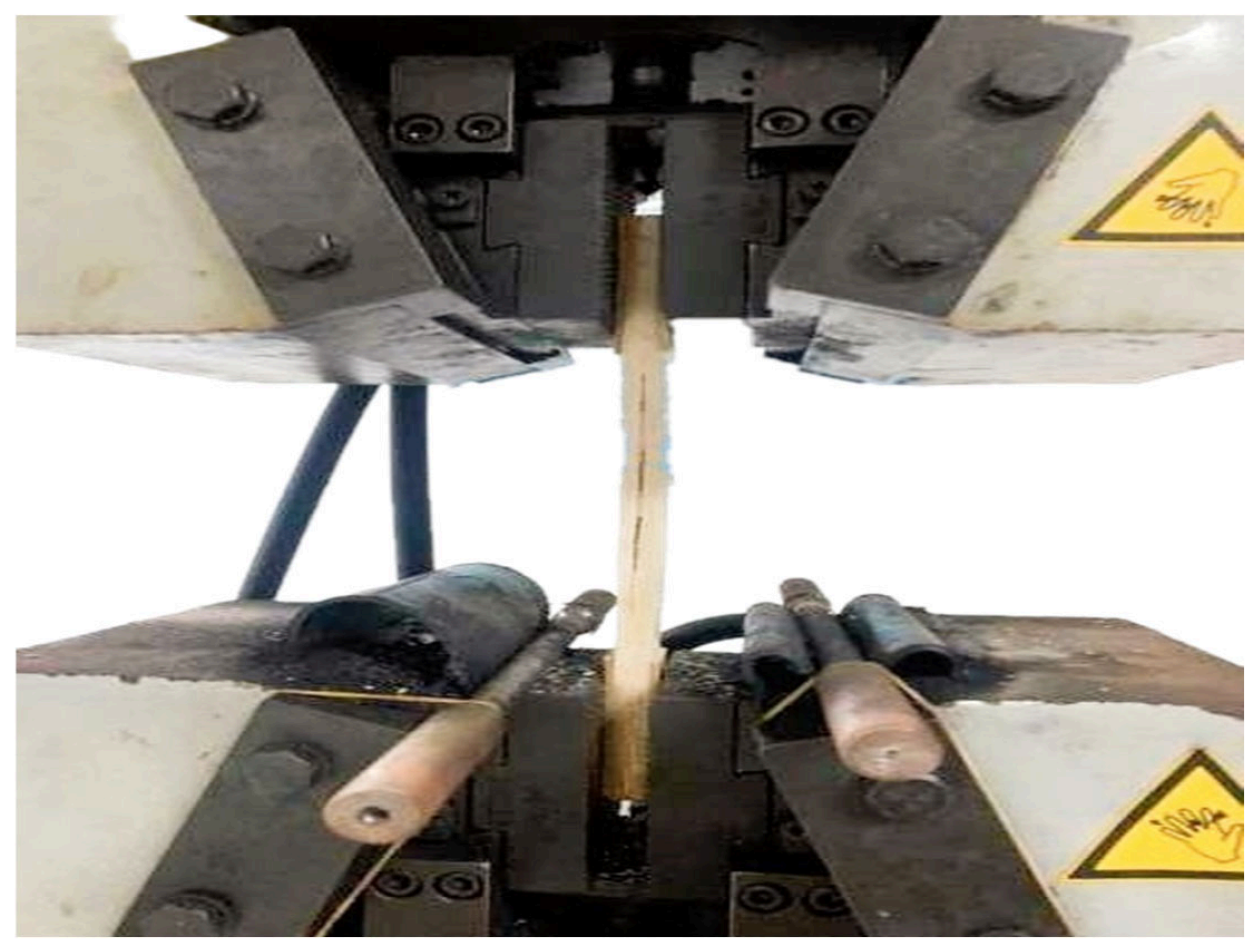
Tensile test device.

The instrument used in this test is a universal testing machine, which adopts the tensile mode of composite materials. The tensile strain at the middle position of the specimen is measured by an extensometer with a range of 50 mm, as shown in Figure 6.

During the loading process, a linear displacement mode was adopted. To enhance the validity of the test data, the loading rate was set at 4 mm/min before reaching 0.2% of the ultimate strength. After reaching the set value, the loading rate automatically decreased to 2 mm/min. Before loading, the storage path, loading frequency, and failure condition of the fiberboard fracture were set. After reaching the failure strength, data collection was completed.

(4) Test contents

The main contents of this test include: the failure states of each specimen; the ultimate bearing capacity of each specimen; the degradation law of tensile properties of specimens; and axial strain of FFRP under different aging times. The entire test process was recorded by photos and text. Through the analysis of the experimental data, the load–displacement curve was further drawn to obtain the ultimate bearing capacity of the specimen. The axial strain was obtained using an extensor with a range of 50 mm. The cross-sectional dimensions of each specimen under different aging times were measured using electronic digital calipers produced by Harbin Measuring Tools and Cutting Tools Group, with specifications ranging from 0 to 150 mm. The accuracy is 0.01 mm.

#### 2.3.3. Interlaminar Shear Test

The interlaminar shear strength (ILSS) of NFRP was evaluated using a modified compression-based procedure with a custom fixture (see Section 2.3.3), rather than the short-beam three-point-bending configuration prescribed by ASTM D2344 [38]. It should therefore be emphasized that the present test is a MODIFIED procedure that deviates from ASTM D2344: the specimens are loaded in uniaxial compression with the custom fixture, so the measured quantity is reported here as the interlaminar shear strength determined under this modified configuration and is used only for relative comparisons; it is not equivalent to the standard short-beam ILSS. The test rate was 1 mm/min and the specimen size was 10 × 10 mm; to improve test reliability, each combination of ply number and aging period contained 5 specimens (i.e., 20 specimens per ply group over the four aging periods). The interlaminar shear test device adopted the processed interlaminar shear steel frame.

(1) Specimen preparation

Cut the specimen to a size of 10 × 10 mm and make marks in the weft direction. Apply a uniform layer of resin glue to the exposed parts of the fibers caused by cutting to prevent the fibers from coming into contact with water in advance during the operation process and breaking prematurely during the test. When applying resin adhesive, the test piece should be picked up with tweezers to prevent the surface of the test piece from being contaminated and affecting its thickness. The specimens coated with resin should be placed in the drying oven for uniform treatment until the resin adhesive has cured.

(2) Specimen design

**Figure 7:**
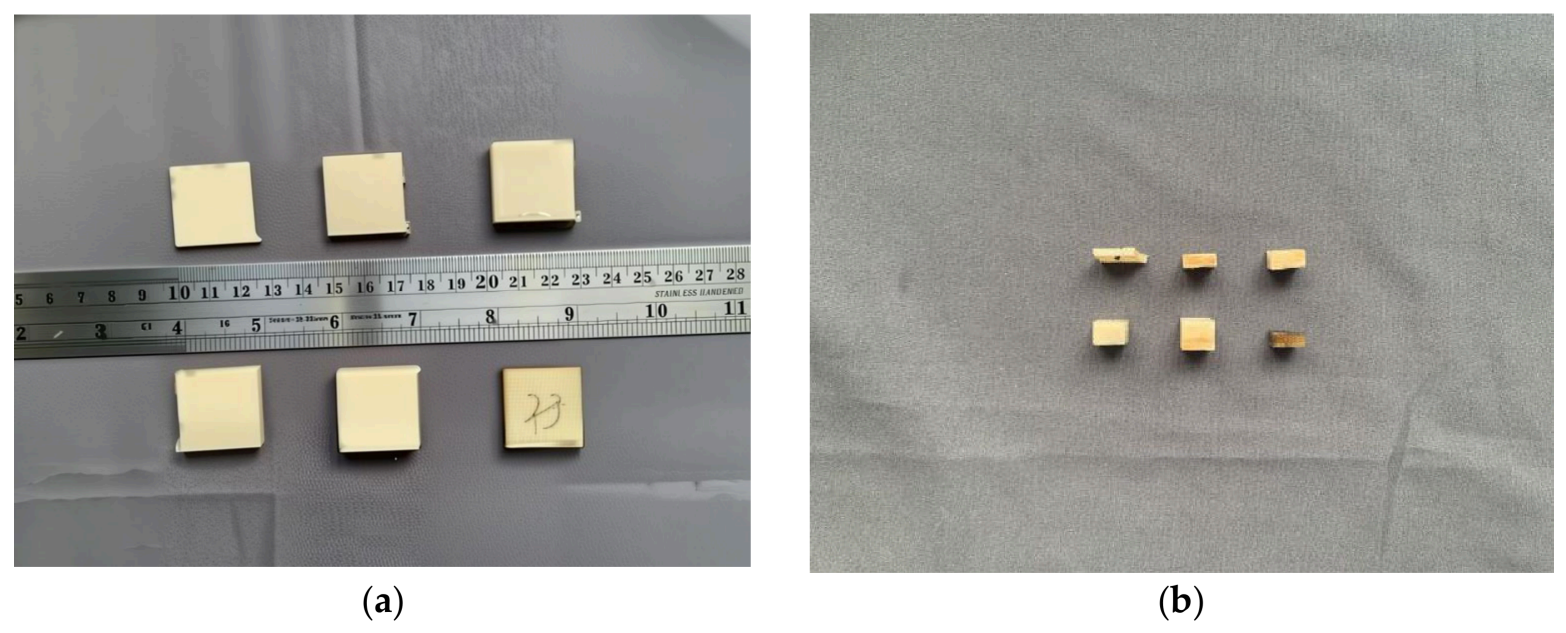
Interlaminar shear specimens. (**a**) Front view of interlaminar shear specimens. (**b**) Side view of interlaminar shear specimens.

The interlaminar shear test was divided into 6 groups, with a total of 120 specimens. Among them, 5 groups were formed by autoclave molding, namely NFRP with 10, 20, 30, 40 and 50 layers of fibers. The other group was the control group, which was made by hand-laid molding of FFRP with a fiber thickness of 10 layers. Each group comprised 20 specimens, i.e., 5 specimens for each of the four aging periods. The test variables are the different numbers of fiber layers, and the different aging times. The naming method of the specimens and the representation of each symbol in the table are the same as those for tensile tests. The specimens are shown in Figure 7.

(3) Test apparatus and loading

**Figure 8:**
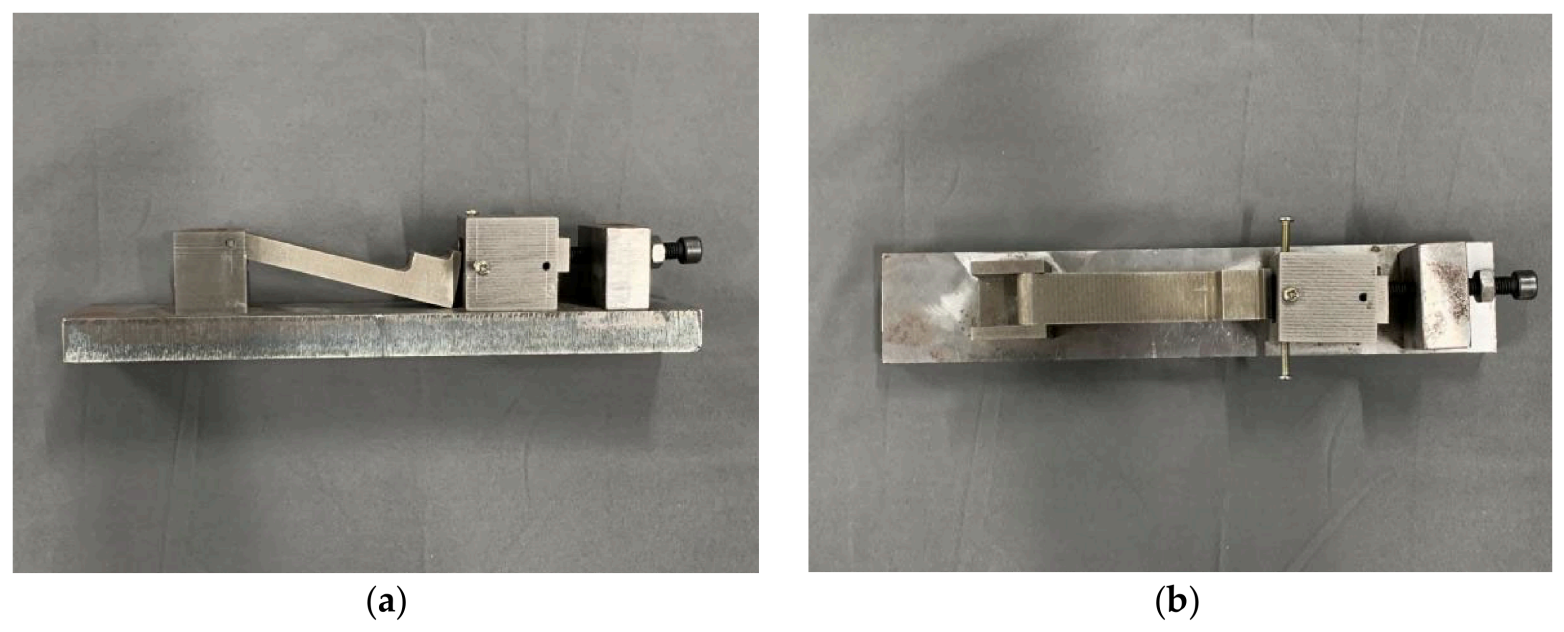
Fixture of interlaminar shear specimens. (**a**) Front view of fixture. (**b**) Top view of fixture.

The instrument used in this test is the universal testing machine produced by Hualong Test Co., Ltd., Shanghai, China, model WAW-1000, and the test mode is uniaxial compression mode. Due to the small size of the specimens, unstable fixation of the specimens occurred. For such on-site conditions, this test improved the original fixture by adding a movable piston at the end where the specimens were fixed, as shown in Figure 8.

**Figure 9:**
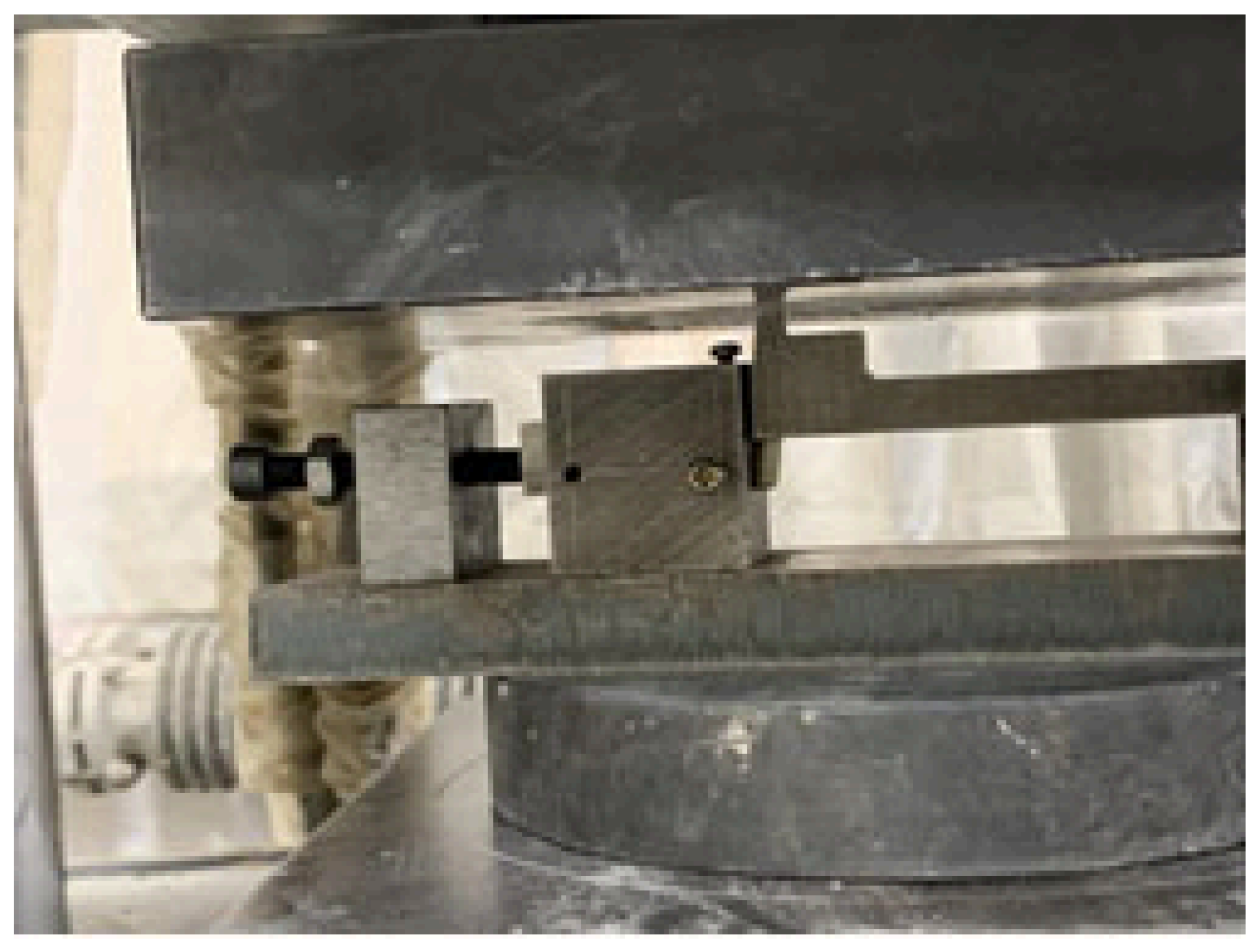
Loading test diagram of interlaminar shear specimen.

During the loading process, a linear displacement mode is adopted. To enhance the validity of the test data, the loading rate is set at 3 mm/min before reaching 0.2% of the ultimate strength. Once the set value is reached, the loading rate automatically drops to 1 mm/min. Before loading, the storage path, loading frequency, and failure condition are set when the specimen reaches 20% of the ultimate strength. Data collection was completed. When installing the specimen, the weft direction should be consistent with the force application direction, and the fixing screws should be tightened. The specimen loading process is shown in Figure 9.

(4) Test contents

The main contents of this test are: the failure states of each specimen; the ultimate bearing capacity of each specimen; the degradation law of shear performance of specimens in a wet and hot environment; and the load–displacement curves of NFRP with the same number of fiber layers under different aging times, as well as the load–displacement curves of NFRP with different numbers of fiber layers under the same aging time.

#### 2.3.4. DMA Test

When conducting thermomechanical property analysis on composite materials, dynamic thermomechanical analysis (DMA) is adopted to determine the elastic modulus of the material and simultaneously test certain characteristic points of the material, such as the glass transition temperature Tg value.

(1) Specimen preparation

**Figure 10:**
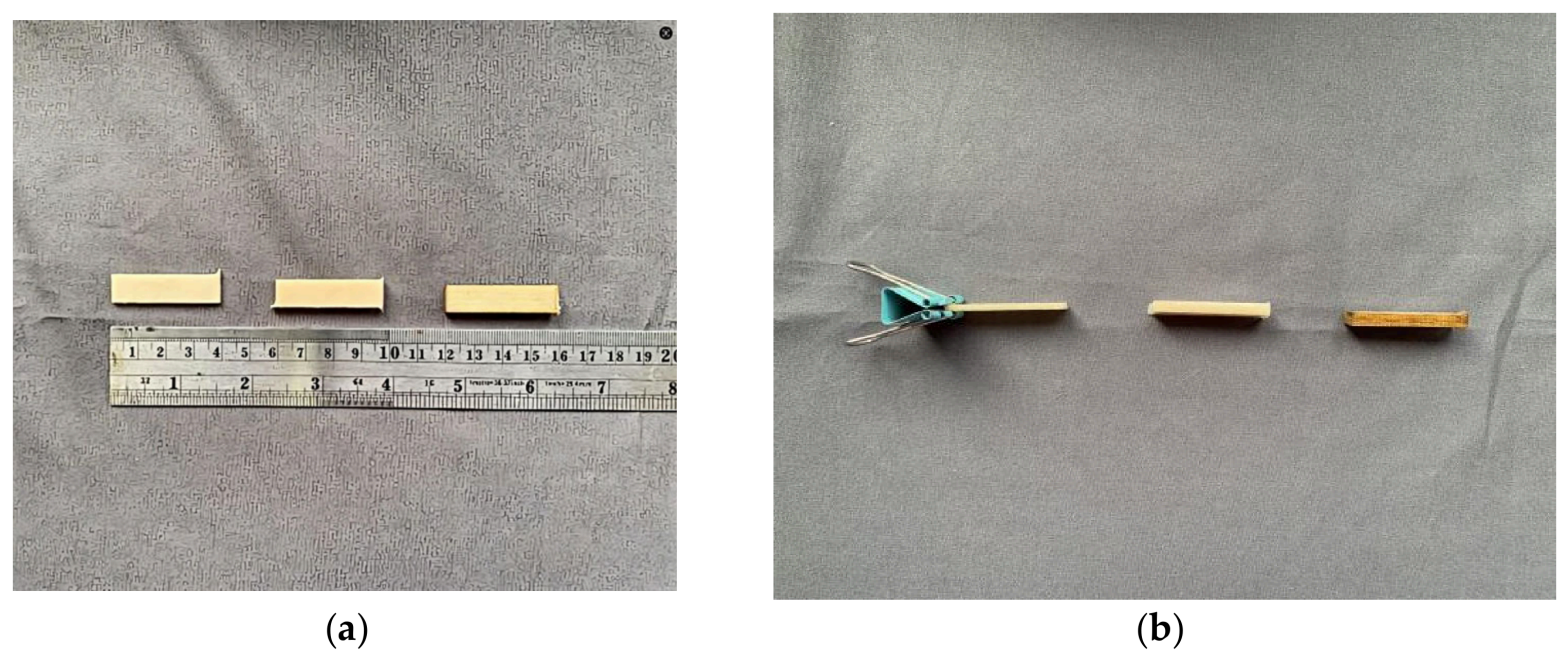
DMA specimens. (**a**) Front view of the DMA specimens. (**b**) Side view of the DMA specimens.

The composite material plates formed by autoclave molding and hand-laid molding were cut into 35 × 10 × t mm. The exposed fiber parts caused by cutting were coated and sealed with resin glue. The remaining action steps were the same as the interlaminar shear test, as shown in Figure 10.

(2) Specimen design

The DMA test was divided into 3 groups, with a total of 84 specimens. Among them, 2 groups were formed by the autoclave molding process, namely FFRP with 10 and 20 layers of fibers, totaling 60 specimens. Another group was the control group. The FFRP was made by hand-laid molding, with a fiber thickness of 10 layers and 24 specimens. The test variables are the different numbers of fiber layers, and the different aging times. The naming method for specimens and the symbols in the table are the same as those for tensile tests.

(3) Test apparatus and loading

Test Device A

Dynamic thermomechanical analysis (DMA) is widely used in the study of viscoelastic properties of materials, and can obtain indicators such as the dynamic storage modulus, loss modulus and tangent of loss angle of materials.

Test B loading

The test used a TA Instruments Q800 dynamic thermomechanical analyzer (TA Instruments, New Castle, DE, USA). The frequency adopted in this test is 1 Hz, the heating rate is 5 °C/min, the temperature range is set from room temperature to 180 °C, and the test mode is single cantilever. The key parameters include an oscillation amplitude of about 20 μm, corresponding to a strain of approximately 0.05%, a preload of 0.03 N, a single-cantilever span of 17.5 mm, and all replicate specimens were used under each condition (10 specimens per condition for the autoclave-molded groups and 8 for the hand-laid group), following ASTM D7028 [39] (Standard Test Method for Glass Transition Temperature of Polymer Matrix Composites by DMA) as the reference standard. It should be noted that the DMA results are reported only up to 15 days, whereas the overall aging cycle can reach 30 days; this is because, as the aging time increases, the material softens markedly through moisture absorption, and the storage modulus and loss factor become flatter with reduced signal stability after 15 days, so DMA characterization was performed only at 0, 7 and 15 days.

(4) Test contents

The three parameters of the DMA test were analyzed. The material composition was reflected by the energy storage modulus E′ of the material, and the loss modulus E″ represented the damping performance of the material. The loss factor (Tan delta) of the material was obtained by the ratio of the loss modulus to the energy storage modulus.

#### 2.3.5. Scanning Electron Microscope Testing

The scanning electron microscope (SEM) is a cold field emission scanning electron microscope produced by Japan Electronics Corporation (JEOL Ltd., Akishima, Tokyo, Japan), with the model number JSM-6700F.

After the 10 layers of water absorption, 50 layers of water absorption and 10 layers of hand-laid tensile and interlaminar shear tests that had not been used and had been used for 30 days respectively, small specimens with a thickness of 1 mm and a length and width of 5 mm were selected and pasted on the iron sheet with double-sided tape.

**Figure 11:**
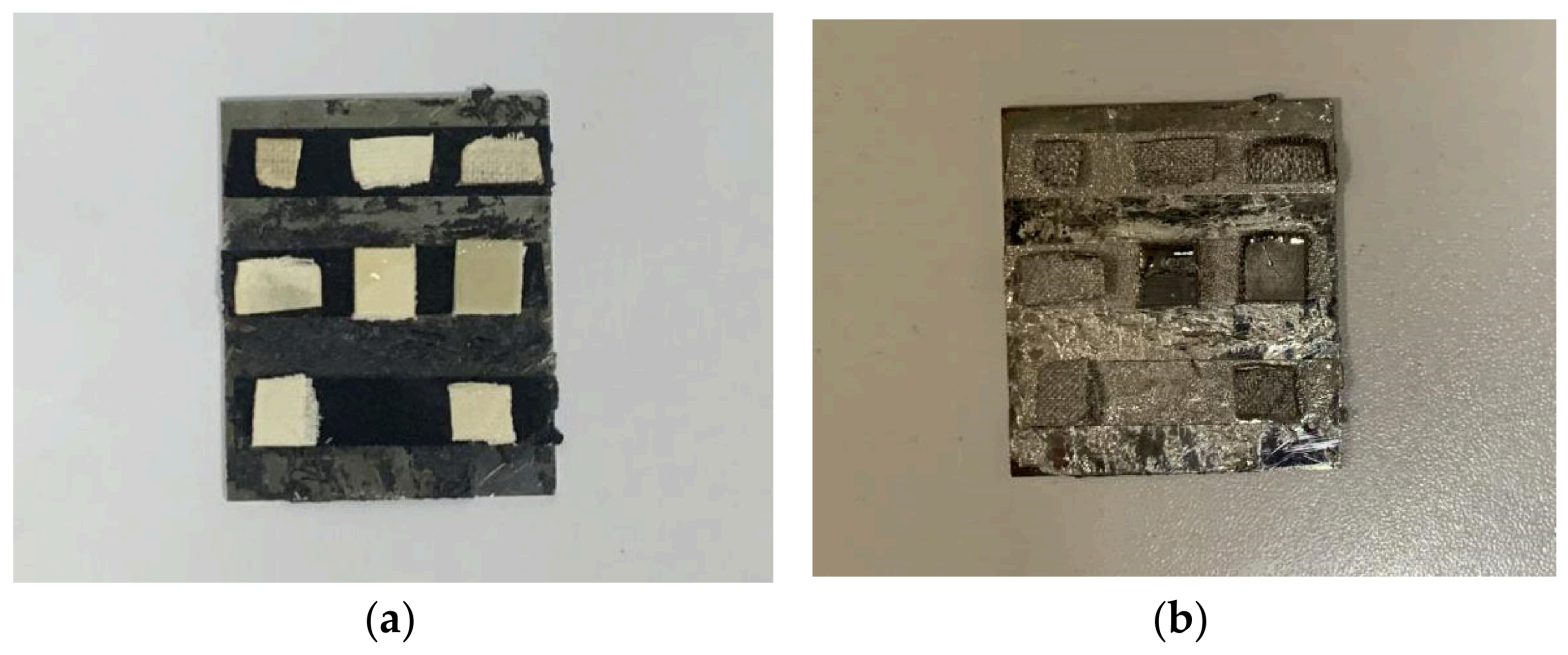
Electron microscope scanning. (**a**) Unprocessed electron microscope scanning specimens. (**b**) Electron microscope scanning images after gold spraying treatment.

The surface of the sample was treated with gold spraying (Ag) for 30 min. As shown in Figure 11, it is the electron microscope-scanned specimen after gold spraying treatment. Secondly, the sample is subjected to vacuum treatment and then observed.

Analysis of tensile and shear mechanical properties of FFRP sheets in a wet and hot environment.

Statistical treatment: all quantitative results are reported as mean ± standard deviation (SD), based on the independent specimens indicated in the test matrix (Table 5). No inferential statistical tests (e.g., ANOVA or *t*-tests) were performed; accordingly, non-statistical quantitative wording such as ‘marked decrease’ or ‘decrease by X%’ is used throughout, and differences are not claimed to be ‘statistically significant’. Error bars (SD) and the corresponding statistics are provided in the figures.

## 3. Results and Discussion

### 3.1. Water Absorption Test

#### 3.1.1. Test Process

**Figure 12:**
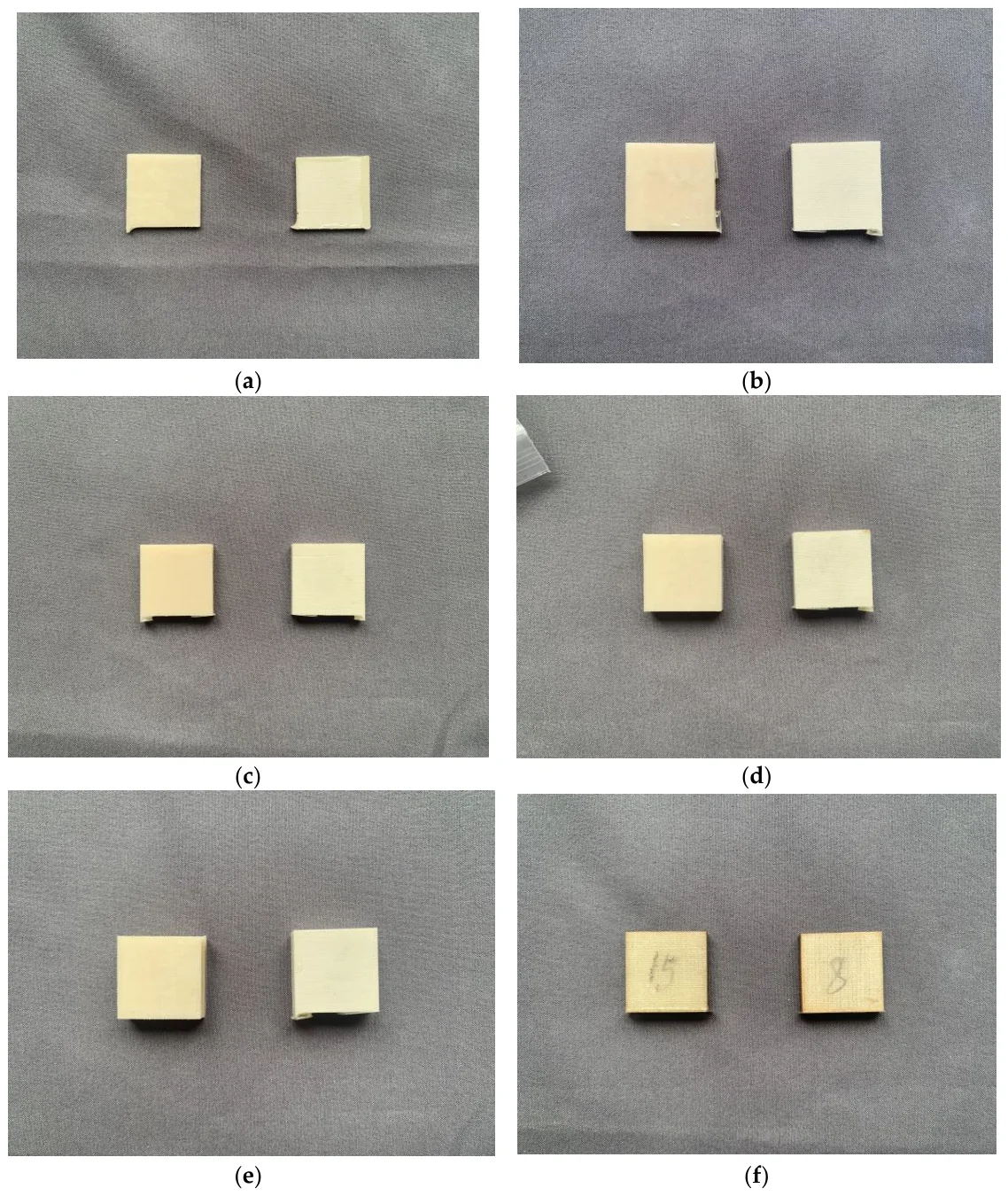
Test comparison of water absorption specimens after curing for 30 days. (**a**) Comparison of water absorption test specimens at 10 layers. (**b**) Comparison of water absorption test specimens at 20 layers. (**c**) Comparison of water absorption test specimens at 30 layers. (**d**) Comparison of water absorption test specimens at 40 layers. (**e**) Comparison of water absorption test specimens at 50 layers. (**f**) Comparison of hand-mixed 10-layer water absorption test specimens.

The water absorption test mainly analyzes the changes in the weight of the sample. Through experimental observation, it was found that as the aging time increased, the surface of the specimens turned white, as shown in Figure 12. The left side of the figure is the specimen that has been treated for 30 days, presenting a white appearance, while the right side is the specimen that has not been treated, presenting a light yellow appearance.

Relevant research indicates that the water absorption performance of composite materials in a humid and hot environment conforms to Fick’s law. When the material’s water absorption reaches saturation, the water absorption rate at this point is the saturated water absorption rate, and the water absorption and diffusion coefficients of the material tend to be in a dynamic equilibrium. At this time, the mass of the material no longer increases.

#### 3.1.2. Diffusion Coefficient

In this paper, the above formula is used to calculate the water absorption rate and diffusion coefficient of FFRP composite materials, and the water absorption performance of the composite materials is obtained. By comparing the diffusion coefficients of specimens with different fiber layers under different aging times, the changes in water absorption performance of each specimen in a wet and hot environment were obtained.

#### 3.1.3. Water Absorption Test Results

As can be seen from the above figure, the water absorption curve of FFRP conforms to the two-stage Fick model in this hot and humid environment. That is, in the initial stage of testing in a hot and humid environment, the mass growth rate of the composite material is relatively large. With the extension of the aging time, the growth rate of the tested specimens gradually decreases.

(1) The water absorption rate of the FFRP board made by hand-laying was similar to that of the 20, 30, 40, and 50 layers in the early stage of the test, but was lower in the later stage, and it had the lowest water absorption rate among all the test groups. The main reason for this phenomenon is that the FFRP board made by the hand-laying process has a lower fiber volume fraction, and the void ratio between fibers and between fibers and branches is also low, so the water absorption rate is relatively low.
(2) The water absorption rate of the 10-layer FFRP plate made by the autoclave molding process was the highest in this test. This paper holds that due to the thinner thickness of 10-layer FFRP plates compared to other specimens, the fiber cloth in the middle layer has a higher participation rate in the test process than that in the middle layer of FFRP with other layers, resulting in a relatively larger water absorption rate for the fiber cloth in the middle layer. Therefore, the water absorption rate of 10-layer FFRP is the highest.
(3) With the increase in the number of fiber layers, the water absorption rate of 10–30 layers of FFRP shows a downward trend under the same aging time. This is consistent with the phenomenon shown in (2). As the thickness increases, the number of fibers in the middle layer participates less in the water absorption test process, resulting in a decrease in the overall water absorption rate of the specimen.
(4) The water absorption rate of the 40- and 50-layer specimens showed a gradually increasing trend in the later stage of the water absorption test. The reason for this phenomenon is that, as the number of fiber layers further increases, the thickness of FFRP increases, and the exposed surface of the fibers in the environment expands, resulting in an upward trend in the water absorption rate. The water absorption performance of each sample was fitted using Equation (1), and the fitting results are shown in Table 6.

As can be seen from Table 6, when the water absorption saturation state is reached, with the increase in the number of fiber layers, the water absorption rate of the composite material as a whole shows an upward trend. The water absorption rate of FFRP composite materials under the 10-layer autoclave molding process is lower than that of the 10-layer FFRP made by the hand-laid molding process. This indicates that the FFRP made by the autoclave molding process is denser than that made by the hand-laid molding process, with fewer gaps between fiber layers, making it difficult for water molecules to enter. Because the thickness of FFRP made by hand-laid molding is approximately 2.4 times that of FFRP made by autoclave molding, the diffusion coefficient of FFRP composite materials with the same 10 layers is relatively high. The 30-layer FFRP exhibits a relatively low water absorption rate, and among FFRP formed by the autoclave process, it has the lowest diffusion coefficient. These observations suggest that at this time, the internal fibers of the composite material appear to be protected by the outer fibers, and in this forming process, the middle-layer fibers are dense, so the water molecule diffusion is low and the water absorption rate is reduced. As the number of fiber layers increases, more voids appear in the middle of the composite materials produced by this autoclave molding process, providing favorable conditions for the diffusion of water molecules. When the number of fiber layers increases, the water absorption rate in the thickness direction of FFRP contributes a relatively large proportion to the result, which is also the reason why the water absorption rate of the composite material with many FFRP layers is relatively high.

**Figure 13:**
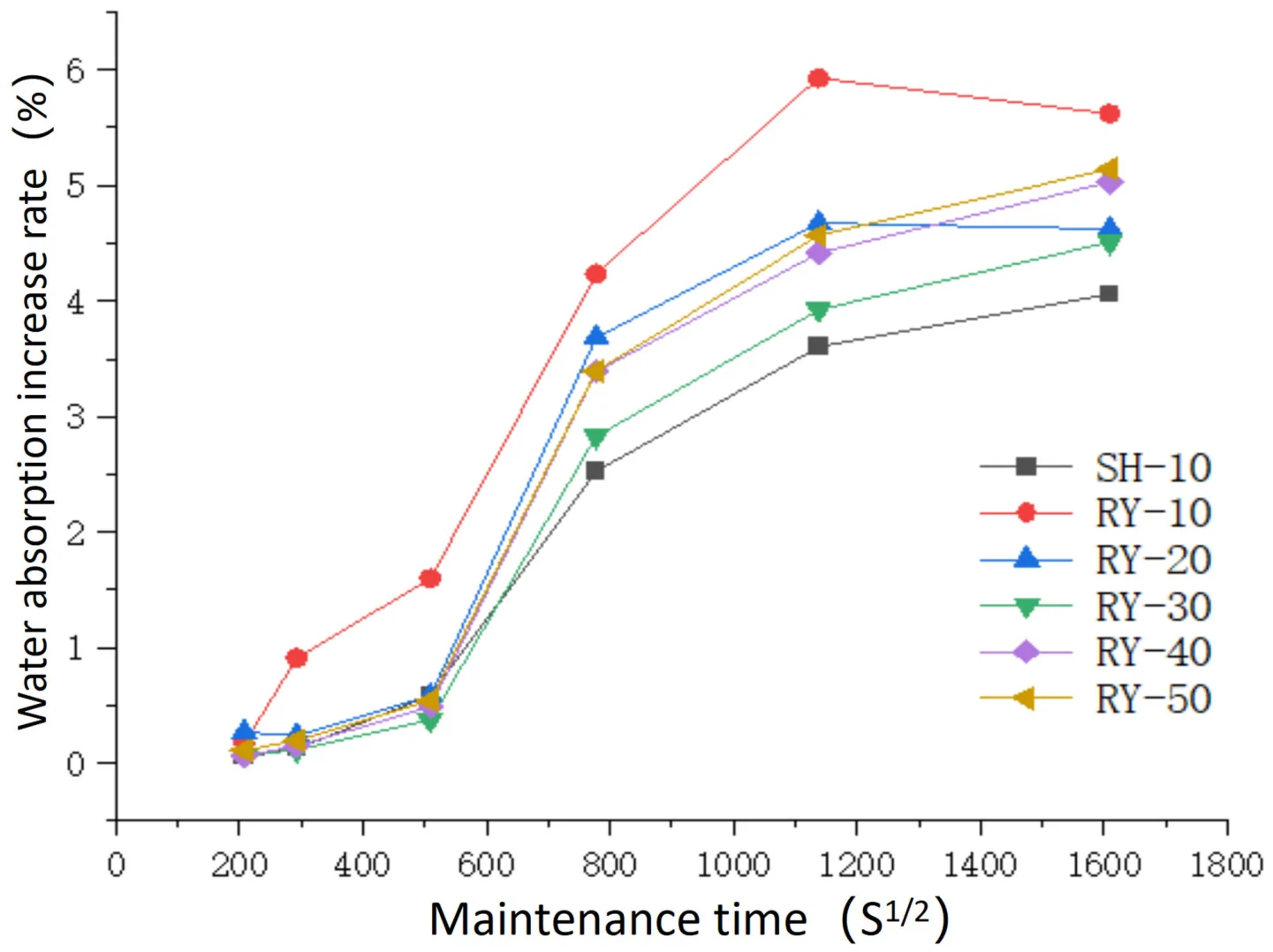
Water absorption curve of the sample during 0–30 days in hot and humid environment.

It should be noted that the fitted saturation water absorption M_m_ in Table 6 is markedly lower than the final water content measured experimentally (for example, the 20-ply specimen reached about 4.5%). This is consistent with the two-stage behavior of the water-uptake curves in Figure 13: Equation (1) is a single-term (single-Fickian) fit, so the obtained M_m_ is only the asymptotic saturation of the Fickian diffusion component and does not include the non-Fickian water uptake induced by the later matrix/interface relaxation. Thus, M_m_ should not be interpreted as the true equilibrium moisture content; together with D, it is used here only as a relative indicator of the diffusion rate and hygroscopic tendency. For the more pronounced later relaxation stage, the single Fickian model has applicability limits, which is acknowledged in the interpretation of the results.

The results of the water absorption test and the fitting results using Fick’s law both indicate that with the increase in the thickness of the fiber layer, the water absorption rate of FFRP first decreases and then increases under the same aging time. When the number of fiber layers is 30, the water absorption rate of FFRP made by the autoclave molding process is relatively low.

In summary, the 30-ply autoclave-molded specimens exhibited relatively low water absorption and diffusion coefficients among the ply numbers tested, but their absorption curves were still rising at 30 days and had not reached a moisture state equivalent to that of the thinner specimens. Therefore, confirming the 30-ply laminate as the preferred configuration is based not only on its lower water absorption but also on its higher tensile/interlaminar shear property retention reported in Section 3.2 and Section 3.3; it should also be noted that thickness differences lead to different diffusion paths, so specimens of different thickness do not reach an equivalent moisture state within the same aging duration, which is taken into account when interpreting the mechanical retention.

### 3.2. Tensile Test

#### 3.2.1. Test Process

**Figure 14:**
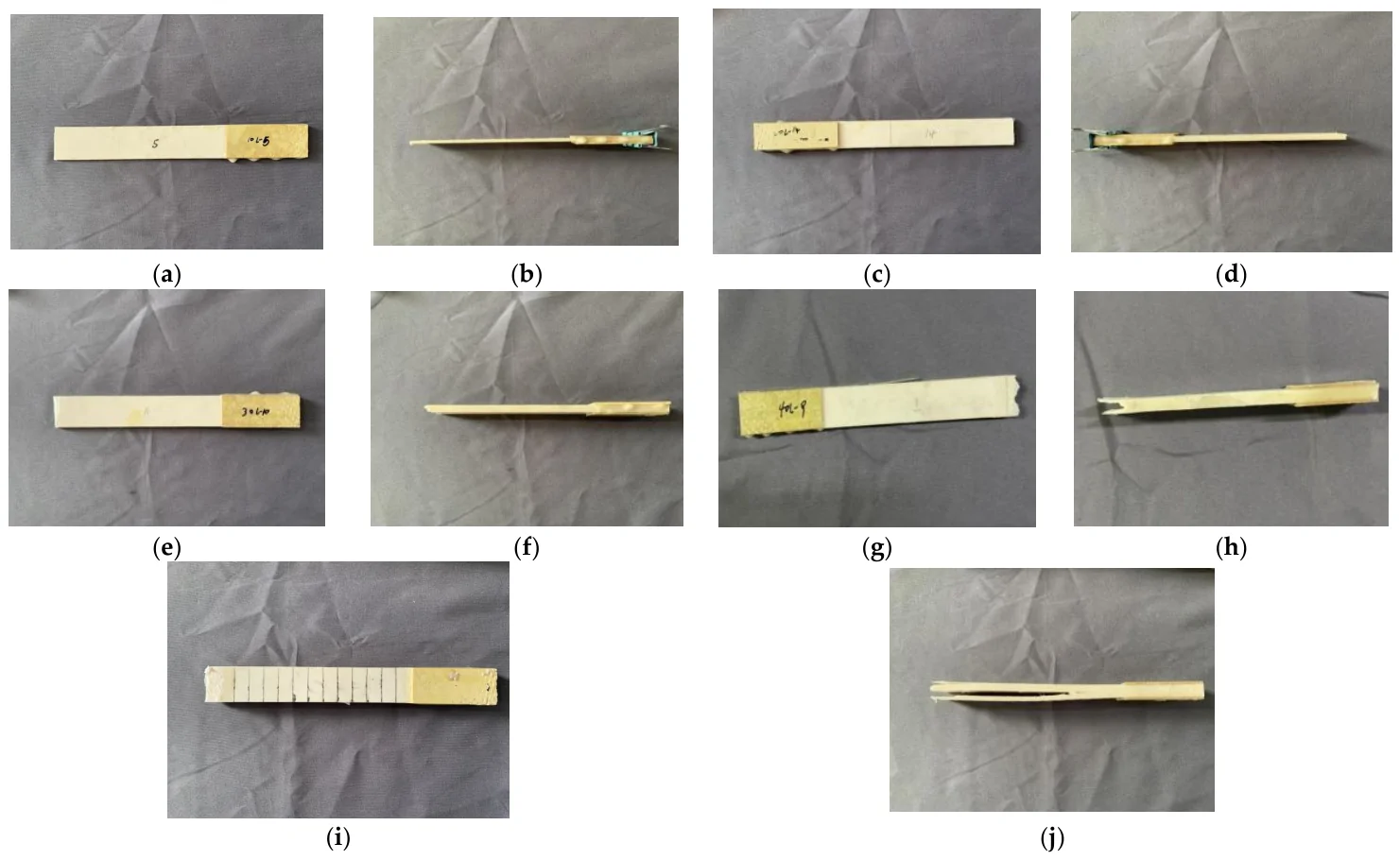
Cross-section breakdown of tensile specimen under 30-day curing. (**a**) Frontal view of the failure of a 10-layer tensile specimen. (**b**) Side view of the failure of a 10-layer tensile specimen. (**c**) Front view of a 20-layer tensile specimen. (**d**) Side view of a 20-layer tensile specimen. (**e**) Front view of a 30-layer tensile specimen. (**f**) Side view of a 30-layer tensile specimen. (**g**) Front view of a 40-layer tensile specimen. (**h**) Side view of a 40-layer tensile specimen. (**i**) Front view of a 50-layer tensile specimen. (**j**) Front view of a 50-layer tensile specimen.

During the tensile test, at the beginning of the loading stage, FFRP shows no obvious symptoms. As the load increases, a relatively crisp tearing sound is produced. With the increase in the load, the FFRP plate suddenly breaks. Figure 14 shows the failure interface diagrams of tensile specimens under the same aging time. Among them, Figure 14a,b are the cross-sectional diagrams of 10-layer tensile specimens with broken rings. The fiber fracture surfaces are neat, and no delamination phenomenon is observed. The specimens on the 10th and 20th layers did not show obvious delamination. The specimens on the 30th layer began to show delamination. The tensile failure sections of the specimens on the 40th and 50th layers showed obvious delamination. It indicates that with the increase in the number of fiber layers, the fracture surface of FFRP gradually shows stratified fracture, the fracture surface presents a serrated shape, and the volume at the fracture site expands. The main reason for this situation is that, as the number of fiber layers increases, interlayer defects between fibers gradually accumulate. When the interlayer defects accumulate to a certain extent, damage clusters are formed and the rings are pre-broken during the force application process. Due to the random distribution of the location and size of the damage clusters, they are interconnected during the failure process, resulting in an uneven, layered and serrated cross-section of the damaged ring. The fracture mode is shown in Figure 14.

**Figure 15:**
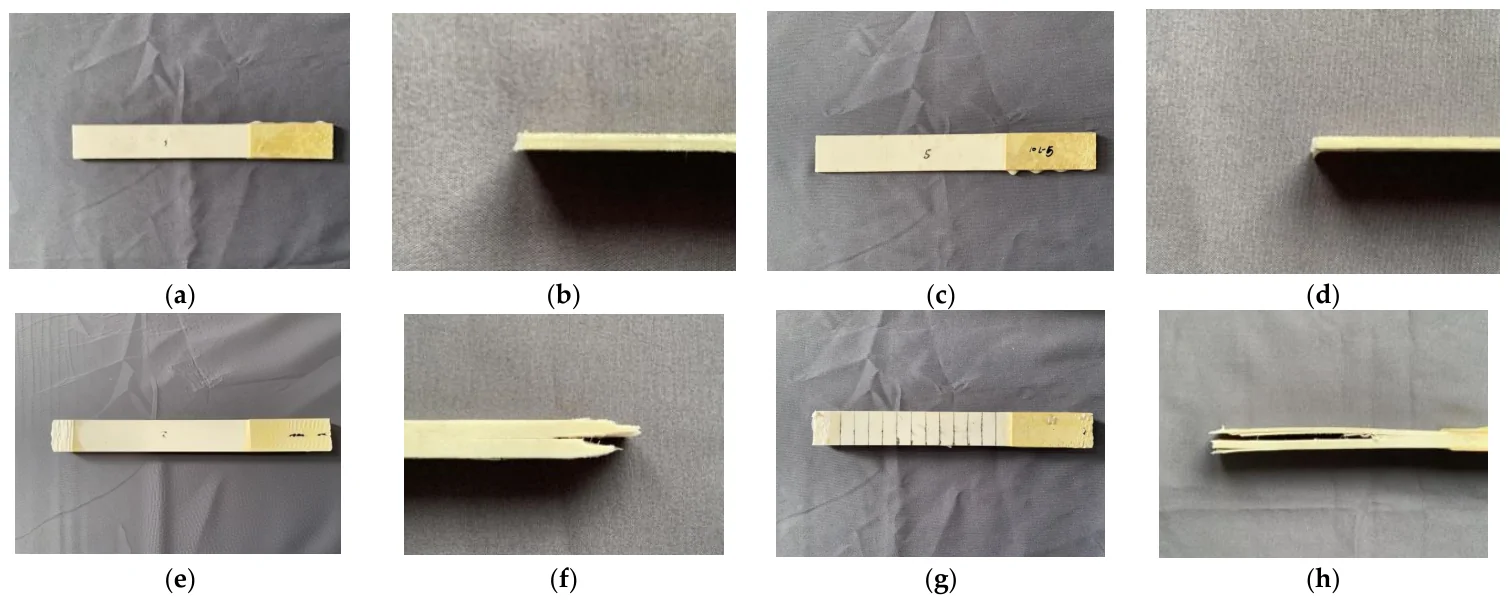
Section diagram of tensile failure under different curing times. (**a**) Frontal view of the failure of a 0 d10-layer tensile specimen. (**b**) Side view of the failure of the 0 d10-layer tensile specimen. (**c**) Frontal view of the failure of a 30 d10-layer tensile specimen. (**d**) Side view of the failure of a 30 d10-layer tensile specimen. (**e**) Frontal view of the failure of the 0 d50-layer tensile specimen. (**f**) Sides of the specimen destroyed by 0 d50-layer stretching. (**g**) Frontal view of the failure of the 30 d50-layer tensile specimen. (**h**) Sides of the specimen destroyed by 30 d50-layer tensile test.

Figure 15 shows the failure cross-section diagrams of the tensile specimens of the 10th and 50th layers at 0 and 30 days of action respectively. It is found from Figure 15a,b that the cross-section of the 10-layer plate does not show delamination under the conditions of no action and 30 days of action, and the cross-section is neat. Moreover, there is no obvious expansion at the fracture position. Figure 15c,d show the failure cross-section diagrams of the 50-layer plate tensile test. By comparing the failure interface of the 10-layer tensile test piece without application, it is found that the 50-layer test piece shows delamination, and the cross-section presents a wavy shape. After 30 days of application, it is observed that the wavy shape of the cross-section of the 50-layer tensile test piece has not further developed, but the delamination phenomenon has intensified. Regardless of whether the 10-layer board was in use or not, no delamination was observed. However, as the application time of the 50-layer board increased, the delamination phenomenon gradually became more obvious. Observation of other specimens revealed that the cross-sections of the 30-layer specimens were inclined, and at this point, the delamination phenomenon of the specimens gradually emerged. Moreover, under the same aging time, as the thickness of the specimens increases, the delamination phenomenon of both 40-layer and 50-layer plates intensifies. Based on the above test phenomena, it is believed that as the aging time and the number of layers increase, the delamination phenomenon of the tensile test failure section becomes more and more obvious. The analysis suggests that as the thickness increases, the number of interlayer defects also increases, which is likely the main reason for the increase in delamination. With the increase in the aging time, due to the existence of interlayer defects, water molecules enter the interior of the specimen more intensively, resulting in the delamination phenomenon of the same specimen gradually becoming obvious.

Due to the differences in forming processes and errors in specimen fabrication, the actual thickness error of FFRP plates is relatively large. By using nominal thickness to reduce experimental errors, the calculation method of nominal thickness is as follows:

(4) $$T_{p}=\frac{m_{au}}{\rho_{v}}$$

In the formula:

T_p_—nominal thickness/mm;

m_au_—mass per unit area/g·mm^−2^;

ρ_υ_—fiber bulk density/g·mm^−3^.

The calculation method for the elastic modulus of FFRP plates refers to ASTM D3039 [37]. The slope of the strain value in the range of 0–1 × 10^−3^ is taken as the elastic modulus of the material.

#### 3.2.2. Tensile Stress–Strain Curve Analysis

**Figure 16:**
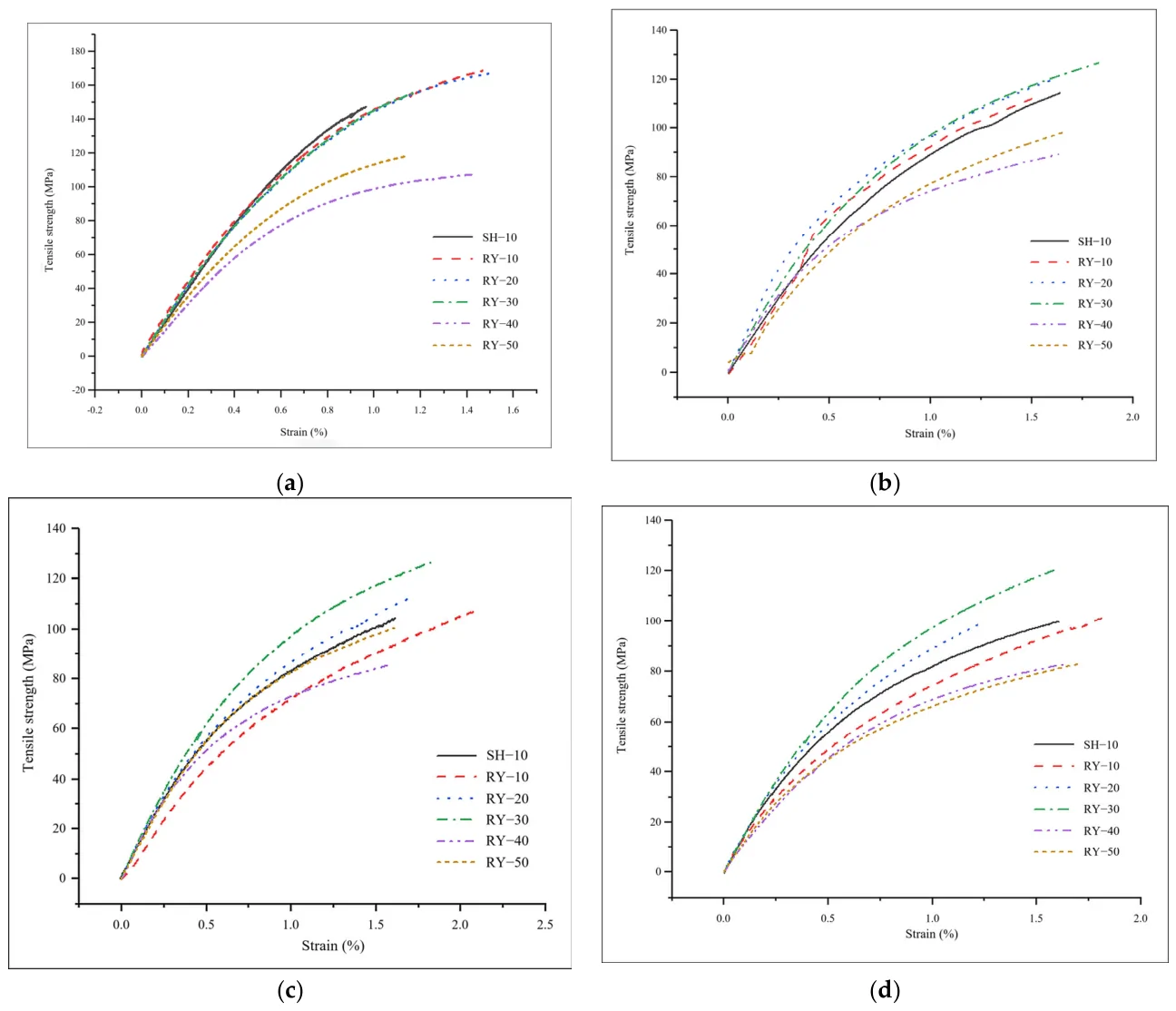
Stress–strain curve of 0–30 d FFRP tensile test in hot and humid environment. (**a**) Tensile stress–strain curve of 0 d FFRP; (**b**) 7-day tensile stress–strain curve of FFRP; (**c**) 15-day tensile stress–strain curve of FFRP; (**d**) tensile stress–strain curve of 30d FFRP.

Existing studies have shown that the stress–strain curve of flax fiber composites exhibits two-stage characteristics. The stress–strain curve of 0 d-FFRP obtained through experiments is consistent with the existing research. As can be seen from Figure 16, after the wet heat test, the stress–strain curve of FFRP shows a gentle shape. The reason for this phenomenon is that as the aging time increases, both the resin and the fibers in the composite material combine with water molecules. Existing research indicates that when flax fibers come into contact with water, they tend to swell between the various wall layers of the fibers, leading to damage to the fibers themselves and debonding at the interface between the fibers and the resin. As the aging time increases, the lignin inside the fibers also degrades, causing the fibers to exhibit greater toughness and a decrease in strength. This is further manifested as a reduction in the tensile strength of the composite material and a more gentle stress–strain curve. The characteristics of the second stage are less obvious than those of the last stage.

#### 3.2.3. Tensile Strength Analysis

The tensile strength of FFRP material is shown in the figure.

**Figure 17:**
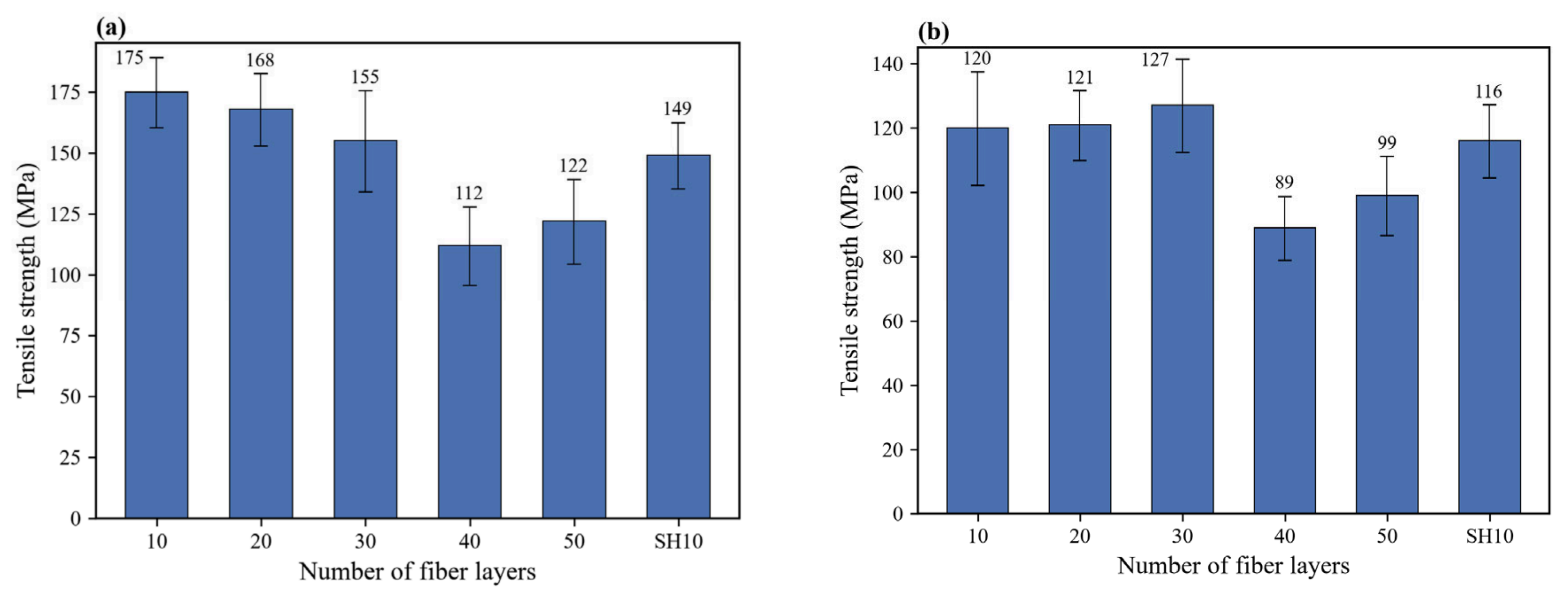

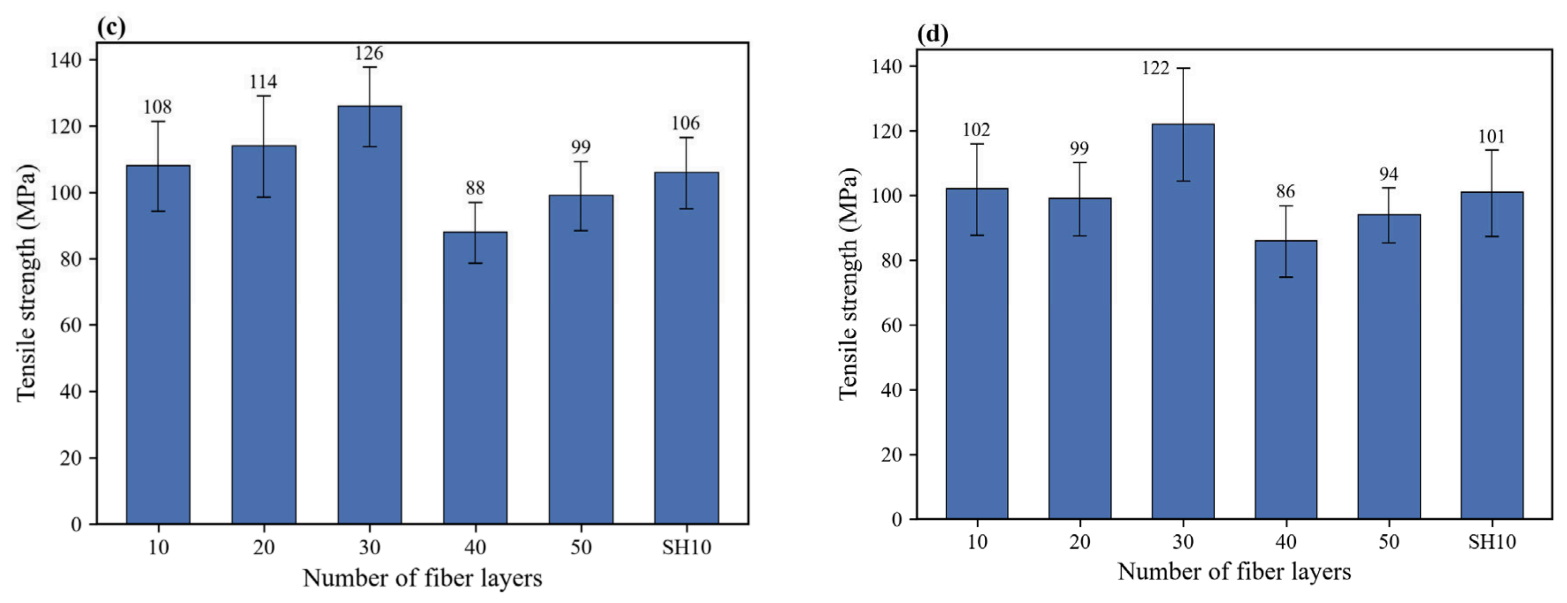
Tensile strength of different specimens under the same curing environment. (**a**) The 0 d environment; (**b**) 7-day action environment; (**c**) 15-day action environment; (**d**) 30-day exposure environment.

Figure 17 shows the tensile strength test under the same aging time. It is observed that in the uncured state, the tensile strength of the specimens first shows a downward trend with the increase in the fiber layer thickness. When it reaches the 40th layer of specimens, the tensile strength no longer increases, and the tensile strength of the 50th layer of specimens slightly increases. These observations suggest that with the increase in the fiber layer thickness, the interlayer defects inside the specimens increase. This leads to an earlier failure during the stretching process; the tensile strengths of the specimens under the 7-day action environment all decreased. Among them, the specimens with 10 and 20 layers had the most marked reduction, reaching 28.11–31.34%, while those with 30 and 50 layers had the lowest reduction, at 17.8% and 18.3% respectively. The analysis suggests that the specimens with a lower number of layers were affected by water molecules, resulting in interlayer damage between fibers and resins. Specimens with a higher number of layers have a lower decrease in tensile strength. The analysis suggests that the outer-layer fibers may provide some protection to the inner-layer fibers, and the influence of water molecules on the middle-layer fibers is relatively small. Observing the test results of 15 days and 30 days, it was found that the tensile strength of the 30-layer fiber specimens was the highest. Under the action of 30 days, the reduction rate of tensile strength was only 21.32%. It was analyzed that the specimens of this number of layers were not only protected by the external fiber layer, but also did not have the accumulation of interlayer defects inside the specimens due to the increase in the thickness of the fiber layer. With the increase in thickness, it was observed that the 40- and 50-layer fiber specimens showed that under the action conditions of 0 d, 7 d, 15 d and 30 d, the decrease in tensile strength was limited because the outer layer of fibers possibly protected the middle layer of fibers. Due to the influence of its forming process and technology, the internal fiber layer is relatively thin, resulting in more internal defects in the specimens, which contribute less to the tensile strength of the specimens.

The analysis of the test results shows that the increase in the thickness of the specimen does not markedly reduce its tensile strength. However, due to the influence of the forming process and technology, its tensile strength does not increase with the increase in fiber thickness in a wet and hot environment.

**Figure 18:**
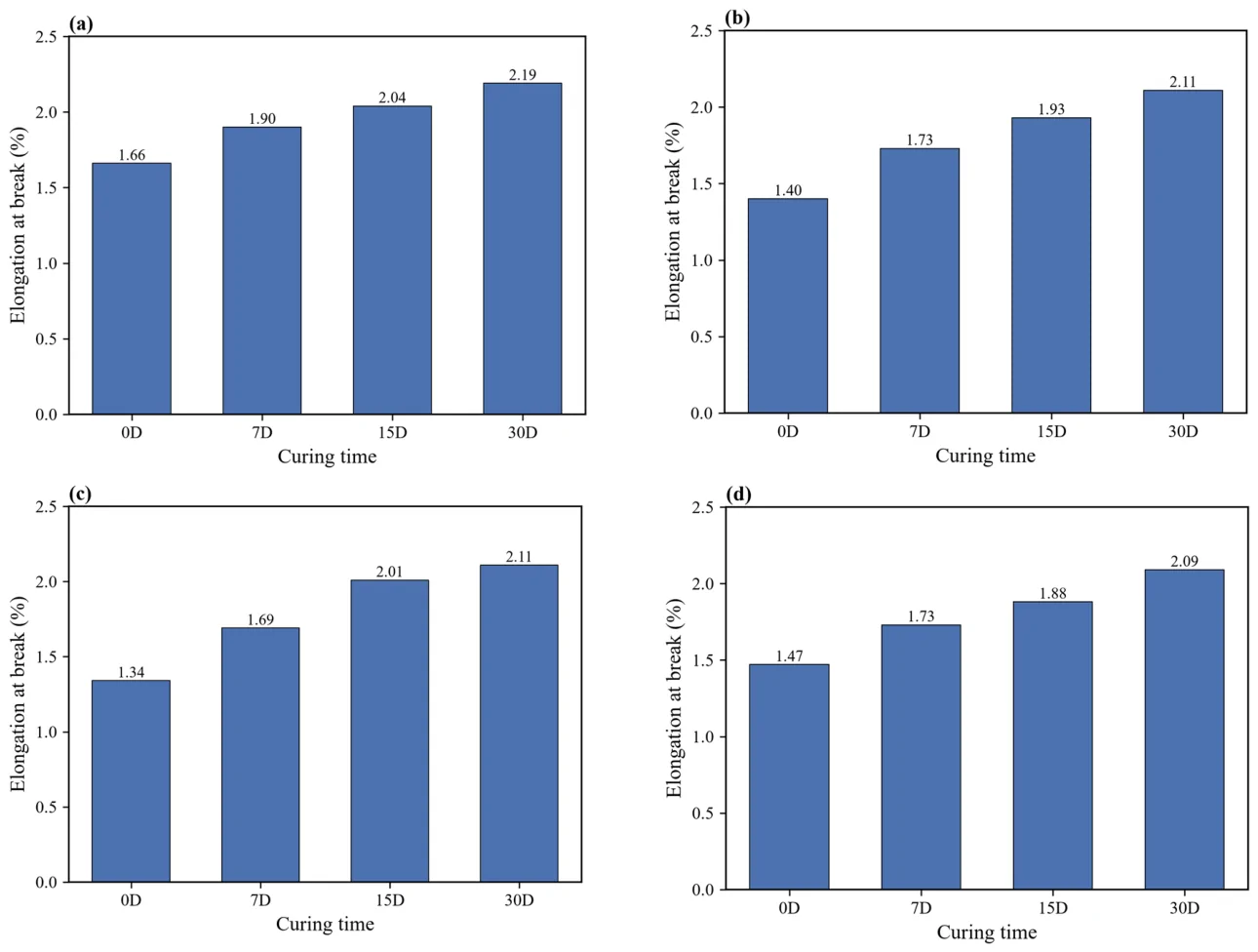

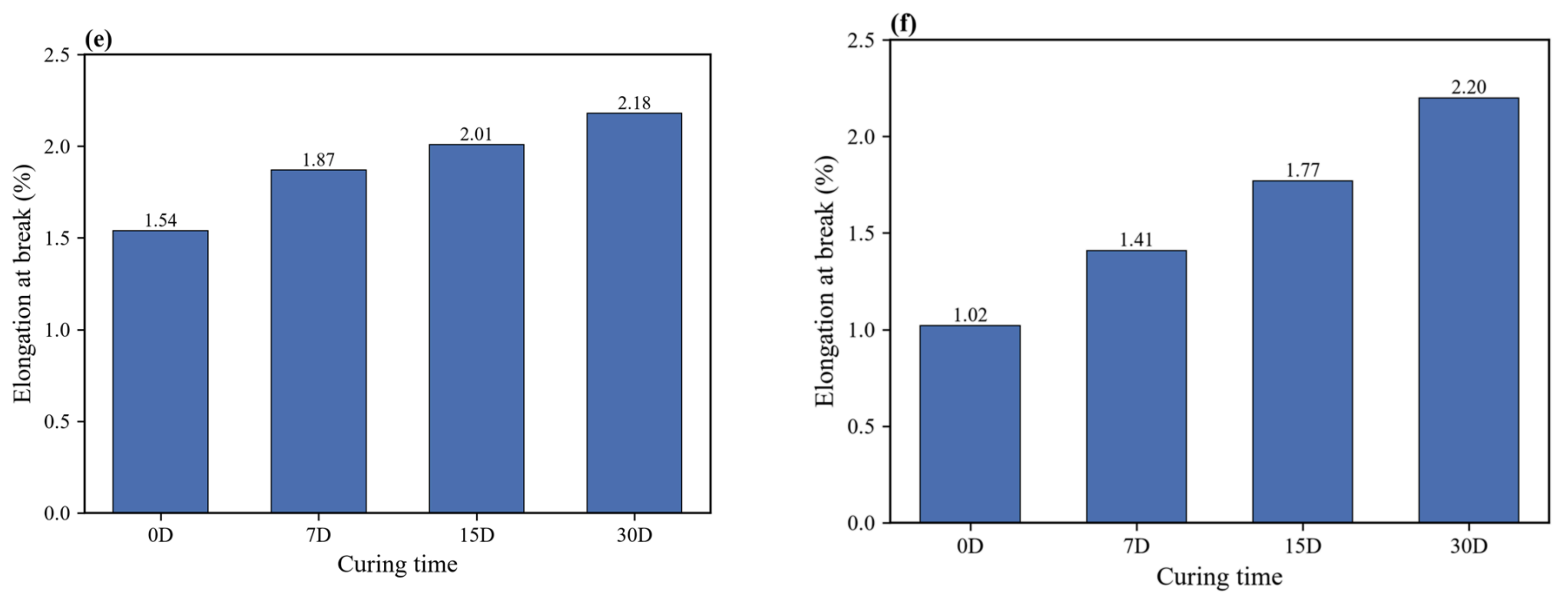
Elongation at break of specimens of each layer. (**a**) Fracture elongation of the 10-layer specimen. (**b**) Fracture elongation of the 20-layer specimen. (**c**) Fracture elongation of the 30-layer specimen. (**d**) Fracture elongation of the 40-layer specimen. (**e**) Fracture elongation of the 50-layer specimen. (**f**) Fracture elongation of the SH10 layer specimen.

Figure 18 shows the changes in the elongation at break of different specimens with the increase in aging time. The elongation at break of all specimens increased with the extension of aging time. The analysis suggests that water molecules disrupted the interface between the resin and the fibers, making the fibers easier to pull out. As the aging time increased, the interface damage became more severe, resulting in a higher elongation at break.

**Figure 19:**
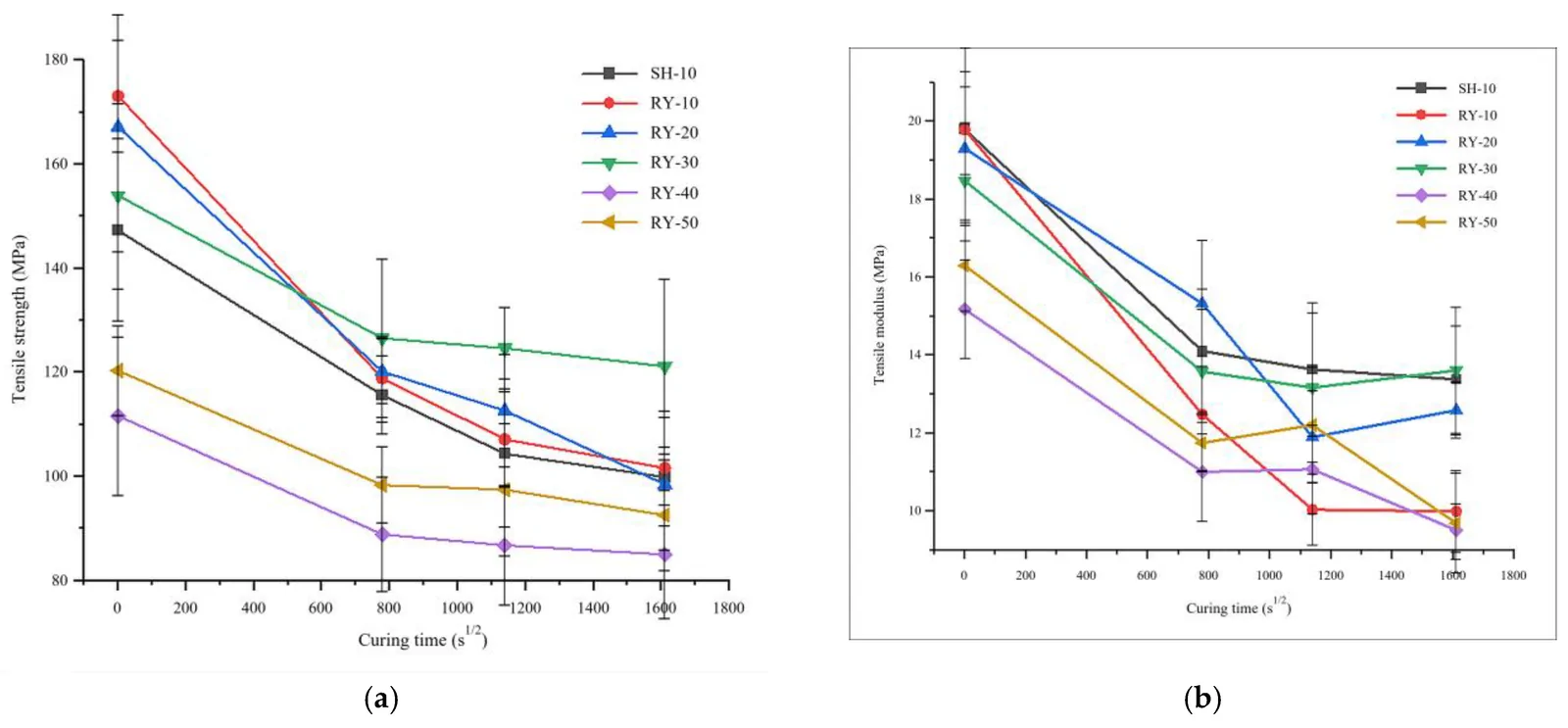
Variation in tensile properties of NFRP. (**a**) Degradation curve of tensile strength of FFRP in humid and hot environment. (**b**) Degradation curve of FFRP tensile modulus in humid and hot environment.

Figure 19 shows that the tensile modulus of flax fiber composites calculated over time also decreases, which is consistent with the existing research results. All indicate that the decrease in tensile modulus is positively correlated with the elongation at break. As can be seen from Figure 19a, the tensile strength shows an overall downward trend. The downward trend in the composite materials made by the two molding processes is most obvious in the 0–7 d environment, and the downward trend in the 10-layer FFRP is the greatest. Under the 7–15 d environment, the measured tensile strength shows a relatively gentle downward trend. However, the FFRP of the hand-laying process and the 10- and 20-layer autoclave process still exhibits a marked downward trend at this stage. Under the conditions of 15 d to 30 d, the downward trend in tensile strength of all test specimens tended to level off. When flax fibers absorb water, their multi-level structure combines with water, causing the microfilaments inside the fibers to rearrange, thereby increasing the microfiber angles and reducing the FFRP tensile modulus.

As can be seen from Figure 19b, the tensile die scales of FFRP produced by the hand-laying process and 10-, 20-, and 30-layer autoclave molding processes show a trend of first decreasing and then increasing. The tensile modulus of 40 and 50 layers of FFRP showed a trend of first decreasing, then increasing, and finally decreasing again under this wet heat test environment, and the final decreasing trend was relatively obvious.

As can be analyzed from the above figure, the tensile strength of FFRP composite materials decreased after being exposed to a damp and hot environment for 30 days. Among them, the decrease in 10 and 20 layers of FFRP was the most marked, reaching 41.2% (±0.11%). Analysis suggests that under this test condition, most of the fibers in the 10 and 20 layers of FFRP were damaged by water absorption due to the relatively small number of external fiber-protective layers, resulting in a marked decrease in tensile strength. The tensile strength of the 30-layer fibers performed the best under this experimental condition, with a decrease of 21.32%. The fibers in the middle layer appeared to be protected by the external fibers. Compared with the 40- and 50-layer FFRP, its interface defects were lower, and it could better exert the advantages and characteristics of multi-layer fibers in tensile tests. With the increase in the number of fiber layers, the elongation at break shows a fluctuating curve of first increasing and then decreasing. As the aging time extends, FFRP with more layers demonstrates better toughness, and the increase in elongation at break is more obvious. Among them, the fracture performance of the 30-layer composite material is the largest, while the 50-layer material had the lowest elongation at break. Analysis suggests that the fibers in the middle layer are protected by the outer-layer fibers, and the interface between the middle-layer fibers and their resin is damaged.

For low-layer specimens, the increase in fiber thickness does not markedly reduce tensile strength. In a wet and hot environment, the tensile strength first increases and then decreases with the increase in thickness. The elongation at break increases with the increase in aging time, and the tensile modulus also shows a downward trend.

### 3.3. Interlaminar Shear Test

Throughout this section, the ‘interlaminar shear strength’/‘interlaminar shear strength’ is the characteristic value measured under uniaxial compression with a custom fixture, rather than the interlaminar shear strength (ILSS) defined by the ASTM D2344 short-beam three-point bending standard. All shear-strength values and retention rates reported below are therefore relative comparisons under this specific configuration and are not cited quantitatively as the standard ILSS.

#### 3.3.1. Material Failure Mode Without Wet Heat Action

**Figure 20:**
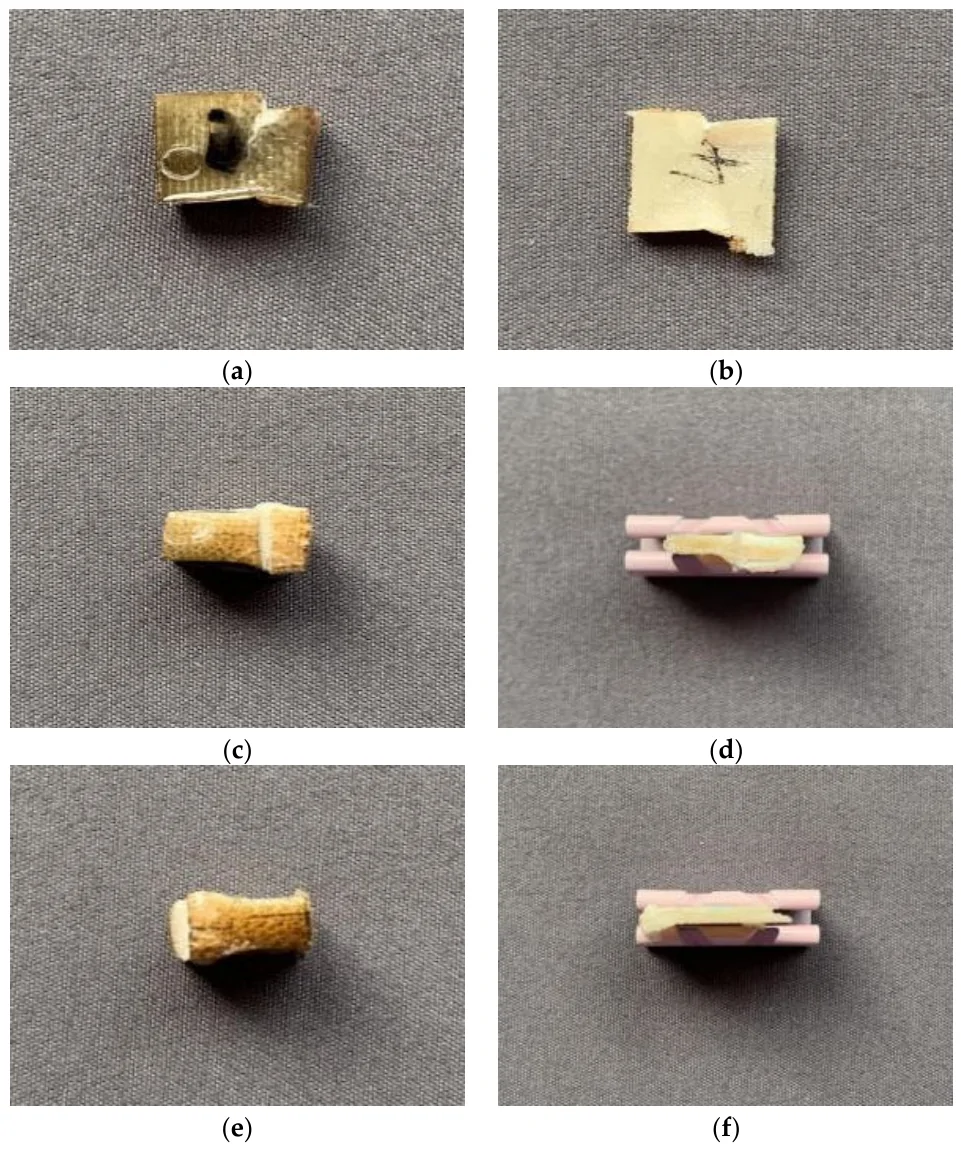
Sectional diagram of interlaminar shear failure of 10-layer specimen without wet heat effect under two processes. (**a**) Hand-pasted 10-layer front view. (**b**) Ten-layer front view. (**c**) Top view of the 10-layer hand-pasted structure. (**d**) Top view of the 10-layer. (**e**) Hand-pasted 10-layer side view. (**f**) Ten-layer side view.

There is no obvious sound during the specimen’s failure process, but a relatively low sound is accompanied by the failure. As shown in Figure 20, it is the cross-sectional view of the interlaminar shear failure test of 10 layers of uncured specimens under two forming processes. The hand-laid 10-layer plate is relatively thick, and no instability failure was found during the loading process, ensuring that shear failure occurred in the force direction of the specimen. The surface cracking was not obvious. No cracks appeared on the side of the force-bearing area. Only the end was subjected to compression failure, which was similar to the failure state of the lower-level specimens. After hot-pressing the 10-layer board, it was found that there were no cracks on the surface, but the force-bearing contact end tilted to one side. The top of the tilted part had delamination, and the cracks were not through. The analysis suggests that the thickness of the specimen is relatively thin. During the loading process of the test, the stability of the force-bearing part cannot be maintained, and instability and failure are prone to occur during the test.

**Figure 21:**
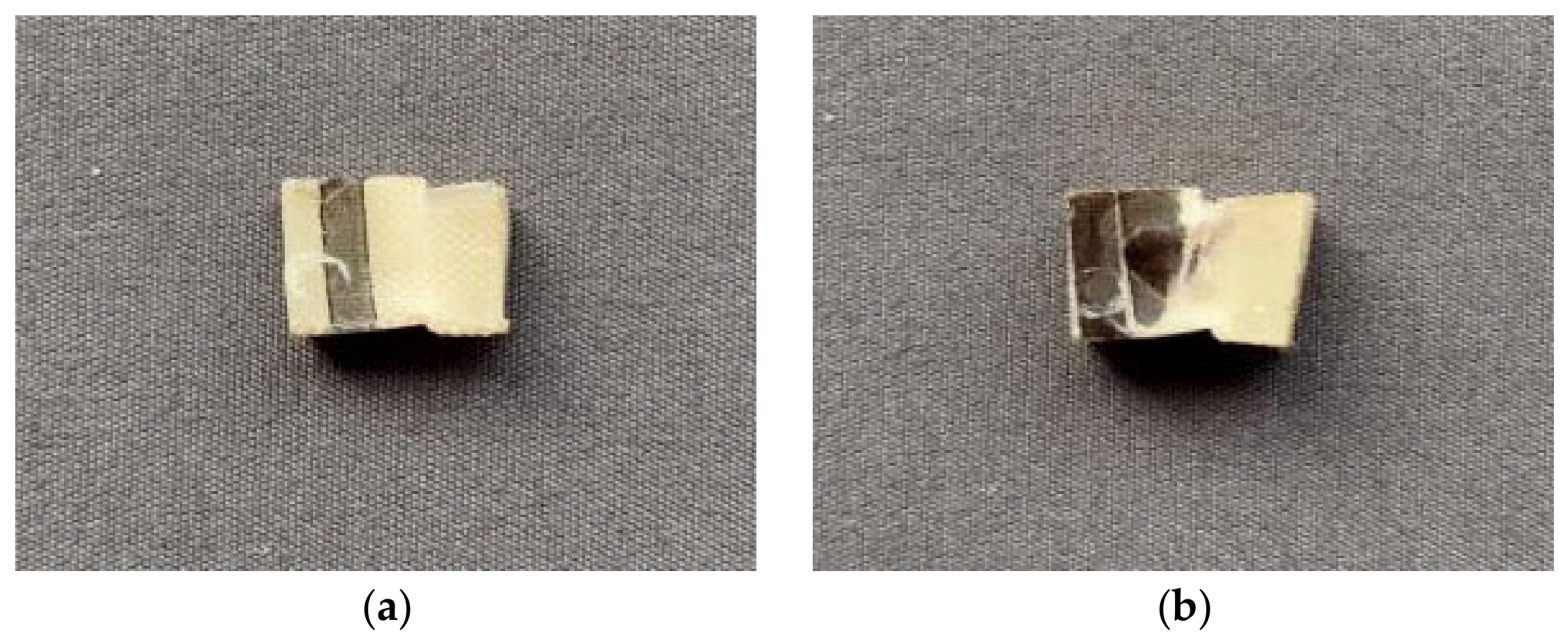

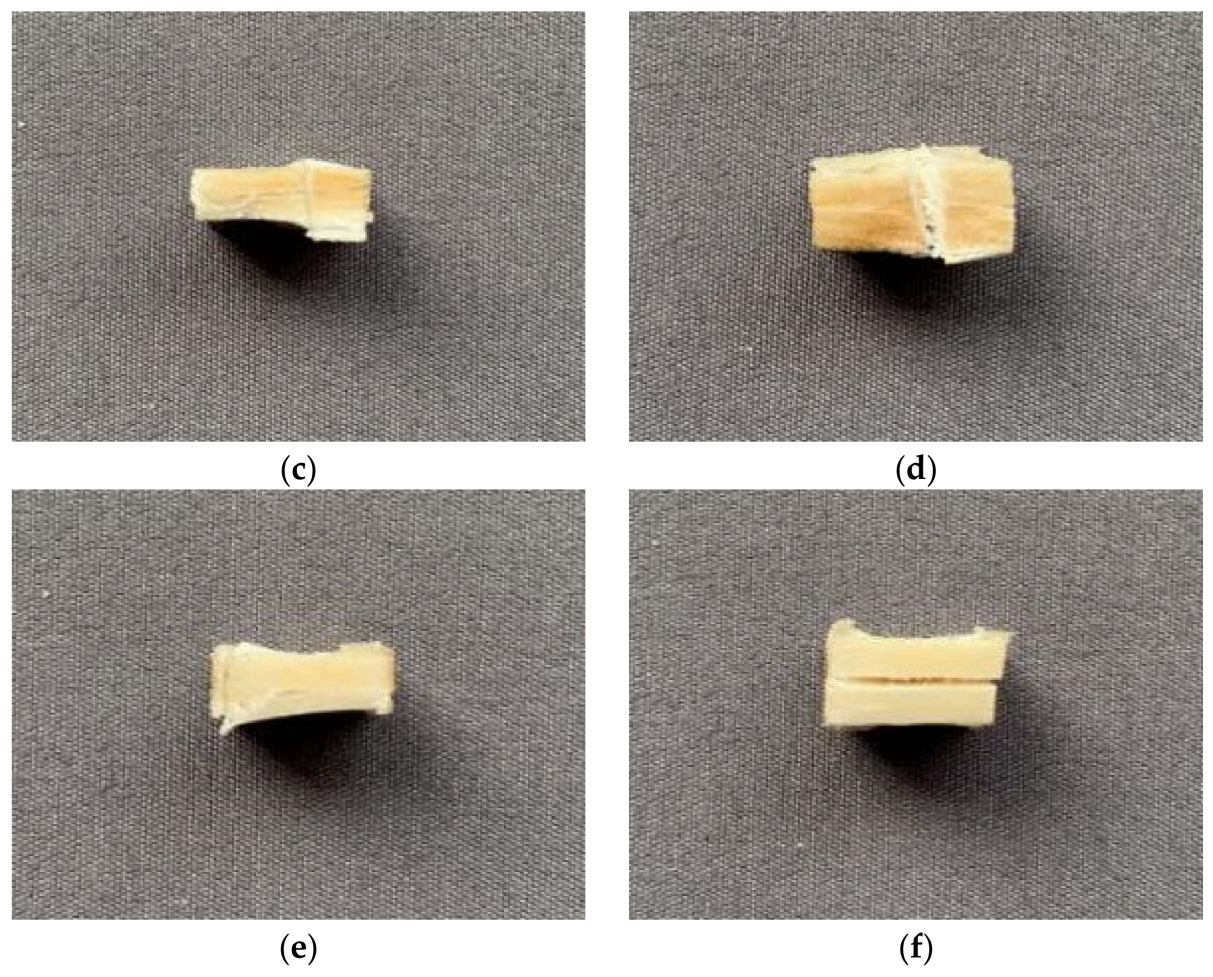
Interlaminar shear failure cross-sections of uncured specimens of 20 and 30 layers. (**a**) Front view of the 20th layer. (**b**) Front view of the 30th layer. (**c**) Top view of the 20-layer specimen. (**d**) Top view of the 30-layer specimen. (**e**) Twenty-layer side view. (**f**) Thirty-layer side view.

As shown in Figure 21, it is the interlaminar shear failure section diagram of the uncured specimens in layers 20 and 30. The surface cracks of the specimen in layer 20 have begun to develop. The stressed end is squeezed and damaged, expanding towards both ends, and the cracks start to develop downward. A through crack formed on the surface of the 30-layer specimen. A distinct crack appeared at the top of the force-bearing area, and there was no obvious expansion at the end. Observing the side view, it was found that the crack was along the force-bearing direction.

**Figure 22:**
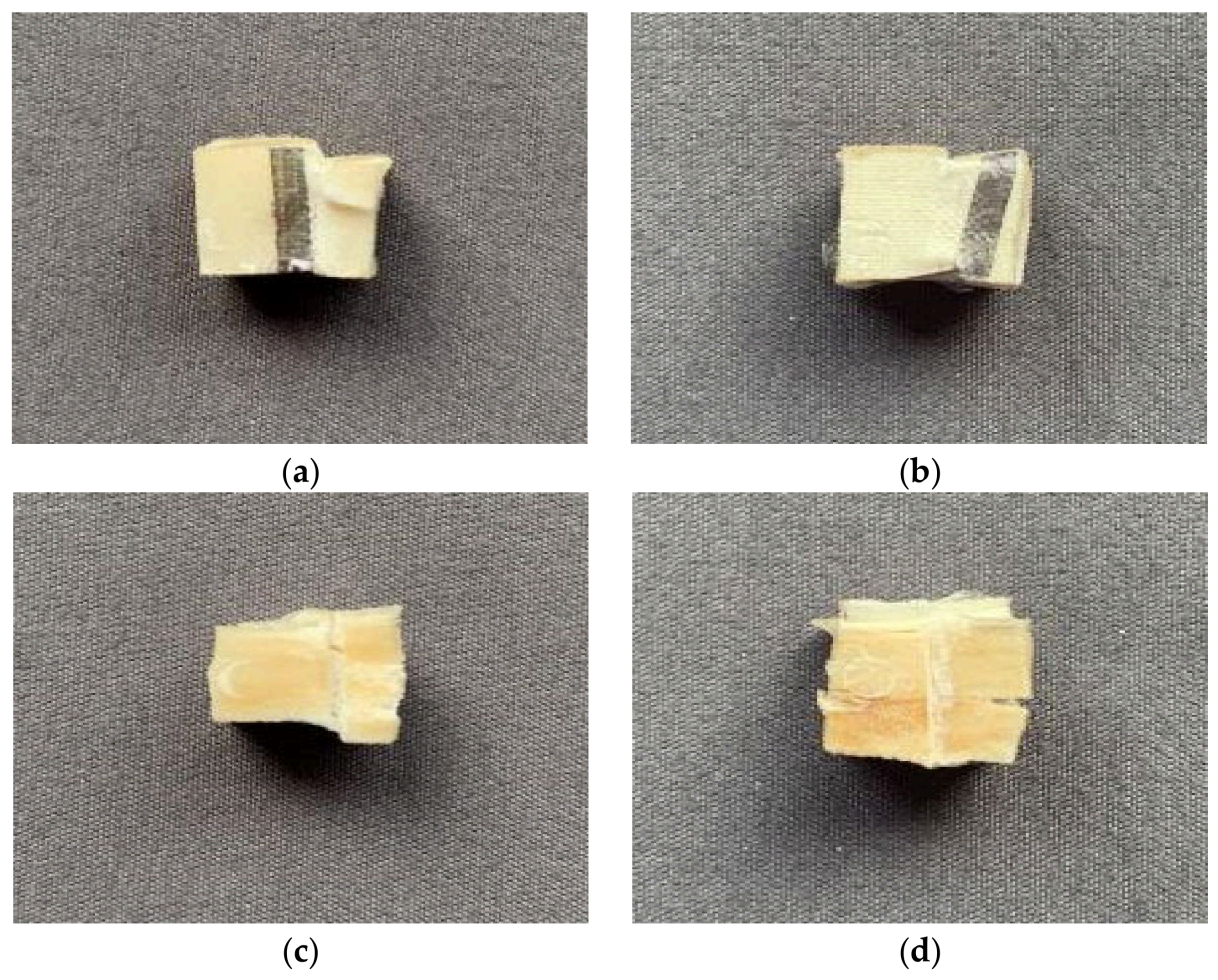

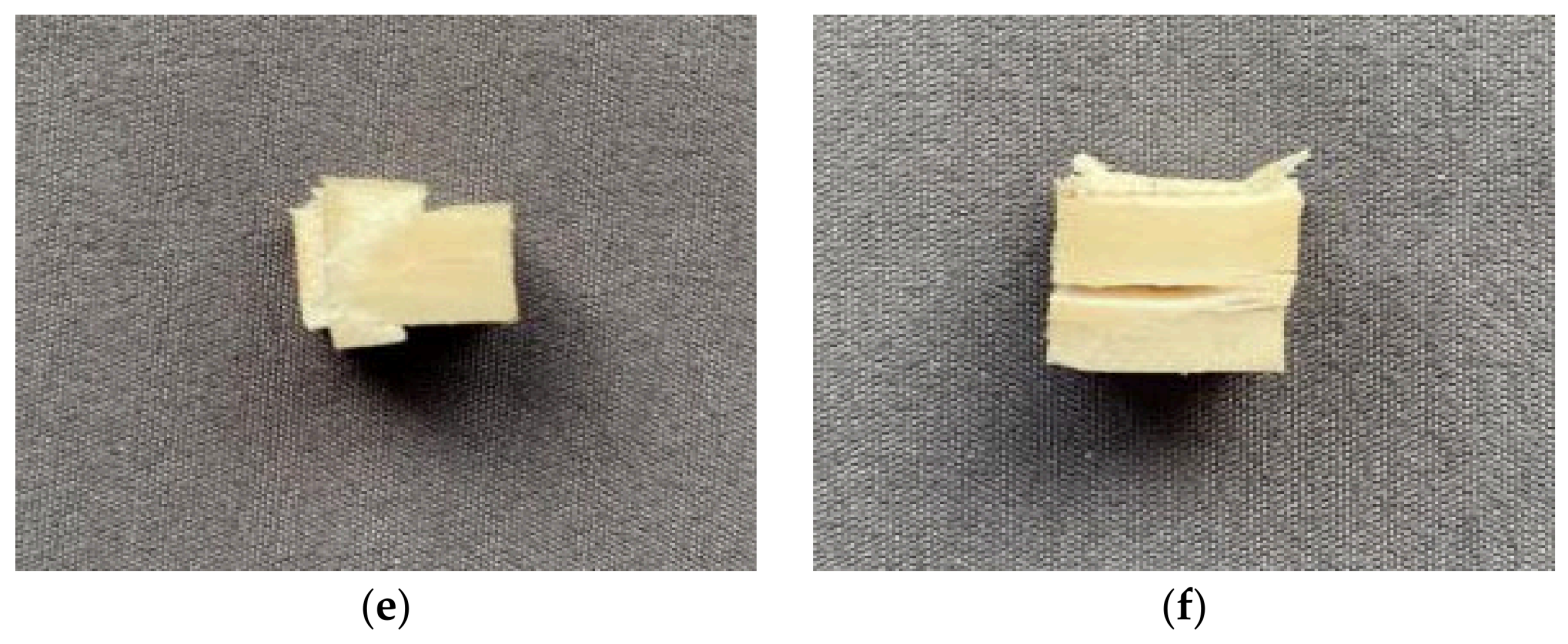
Cross-section of interlaminar shear breakage of 40- and 50-layer uncured specimens. (**a**) Front view of 40-ply laminate. (**b**) Front view of 50-ply laminate. (**c**) Top view of 40-ply laminate. (**d**) Top view of 50-ply laminate. (**e**) Side view of 40-ply laminate. (**f**) Side view of 50-ply laminate.

Figure 22 shows the interlaminar shear failure cross-section diagrams of the 40th and 50th layers of uncured specimens. The surface crack development of the 40-layer specimen is not obvious, but the fiber expansion at the acceptance point is relatively prominent, and there are signs of cracking at the top of the clamping end. The 50-layer test surface did not form through cracks, but the compression zone was clearly stratified, forming a large through crack, surrounded by other tiny cracks.

#### 3.3.2. Comparison of Failure Modes Between Uncured and 30-Day-Cured Specimens

**Figure 23:**
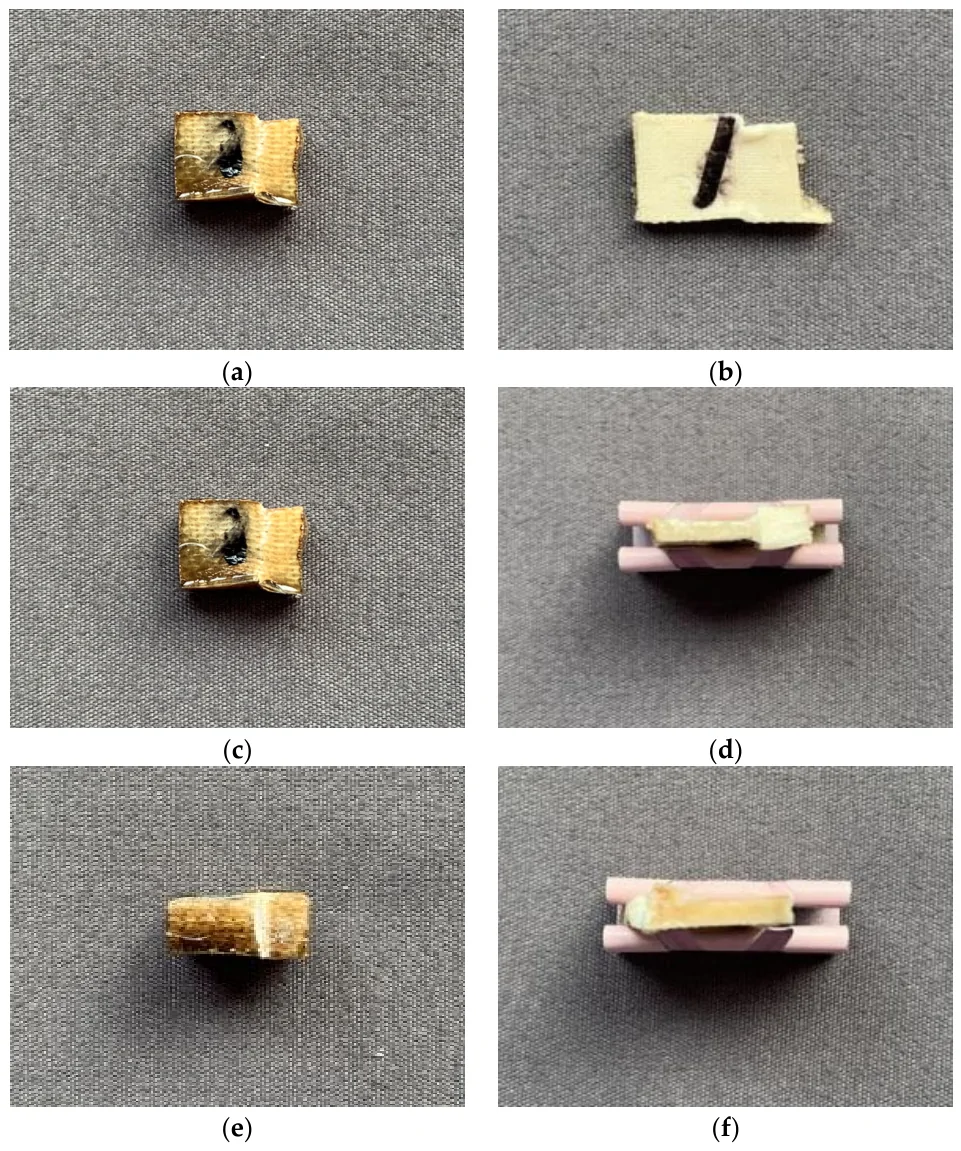
Section diagram of interlaminar shear breakage of 30 d specimen cured with 10 layers under two processes. (**a**) Front view of hand-laid 10-ply laminate. (**b**) Front view of 10-ply laminate. (**c**) Top view of hand-laid 10-ply laminate. (**d**) Top view of 10-ply laminate. (**e**) Side view of hand-laid 10-ply laminate. (**f**) Side view of 10-ply laminate.

As the duration of action increases, the sound at which the fracture starts gradually decreases, but it remains deep. Figure 23 shows the cross-sectional views of interlaminar shear failure of 10 layers of specimens under two processes for 30 days. Through cracks formed on the surface of the hand-laid 10-layer board, while no cracks or top compression failure were observed in the compression zone, indicating that the failure was mainly caused by surface cracks. There were no obvious cracks on the surface of the 10-layer specimens. The failure was mainly caused by the failure of the compression zone at the end. The top of the compression zone uniformly expanded to both sides, and no obvious cracks occurred.

**Figure 24:**
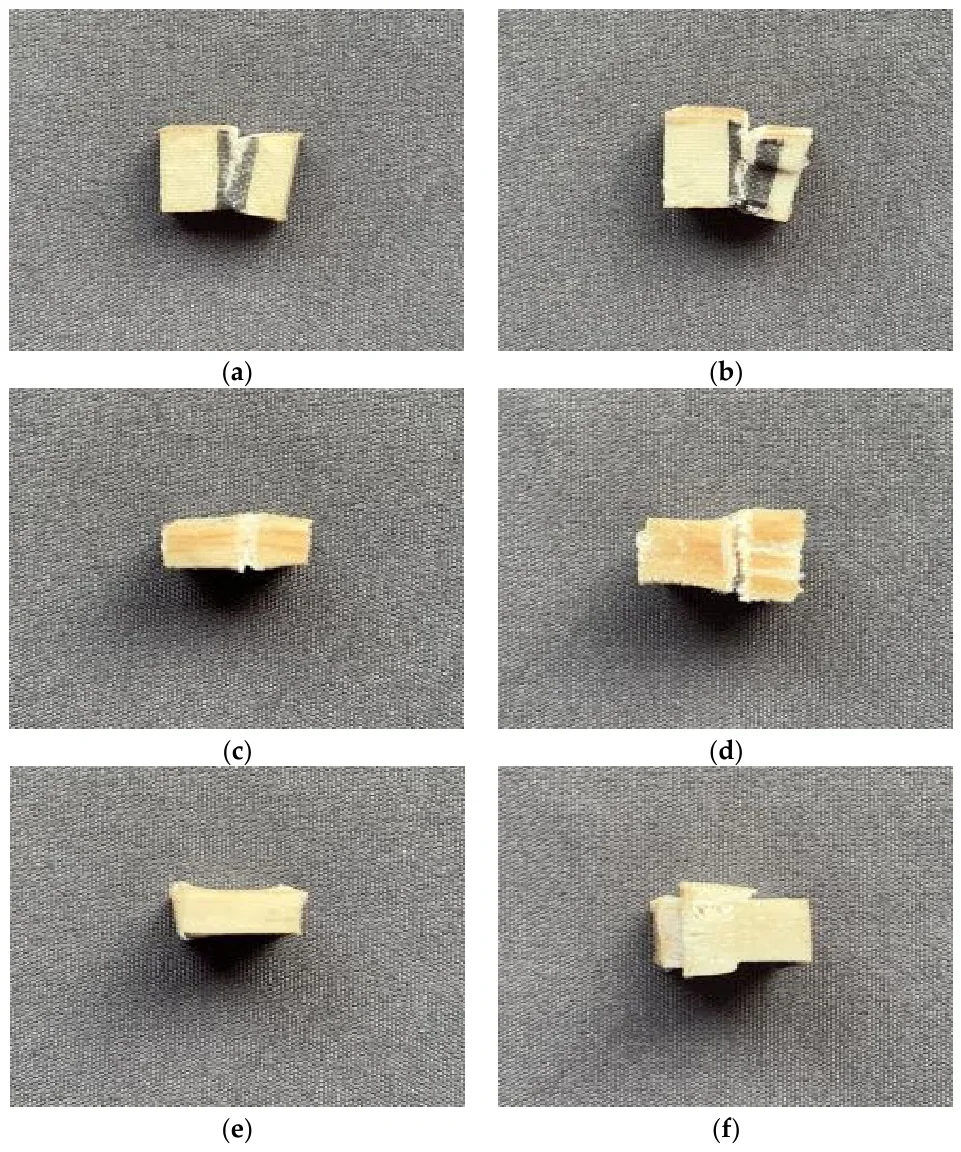
Interlaminar shear failure cross-sections of 30d specimens cured with 20 layers and 30 layers. (**a**) Front view of 20-ply laminate. (**b**) Front view of 30-ply laminate. (**c**) Top view of 20-ply laminate. (**d**) Top view of 30-ply laminate. (**e**) Side view of 20-ply laminate. (**f**) Side view of 30-ply laminate.

Figure 24 shows the interlaminar shear failure of the 20-layer and 30-layer specimens after 30 days of action. The surface of the 20-layer specimen has through cracks, and there is no obvious expansion deformation in the compression zone. The failure of the specimen is caused by the through cracks on the surface. The through cracks on the surface of the 30-layer specimens further developed. The surface of the compression zone was deformed due to the top compression, and the internal delamination was severely damaged at the compression top. There were no obvious cracks at the lower end.

**Figure 25:**
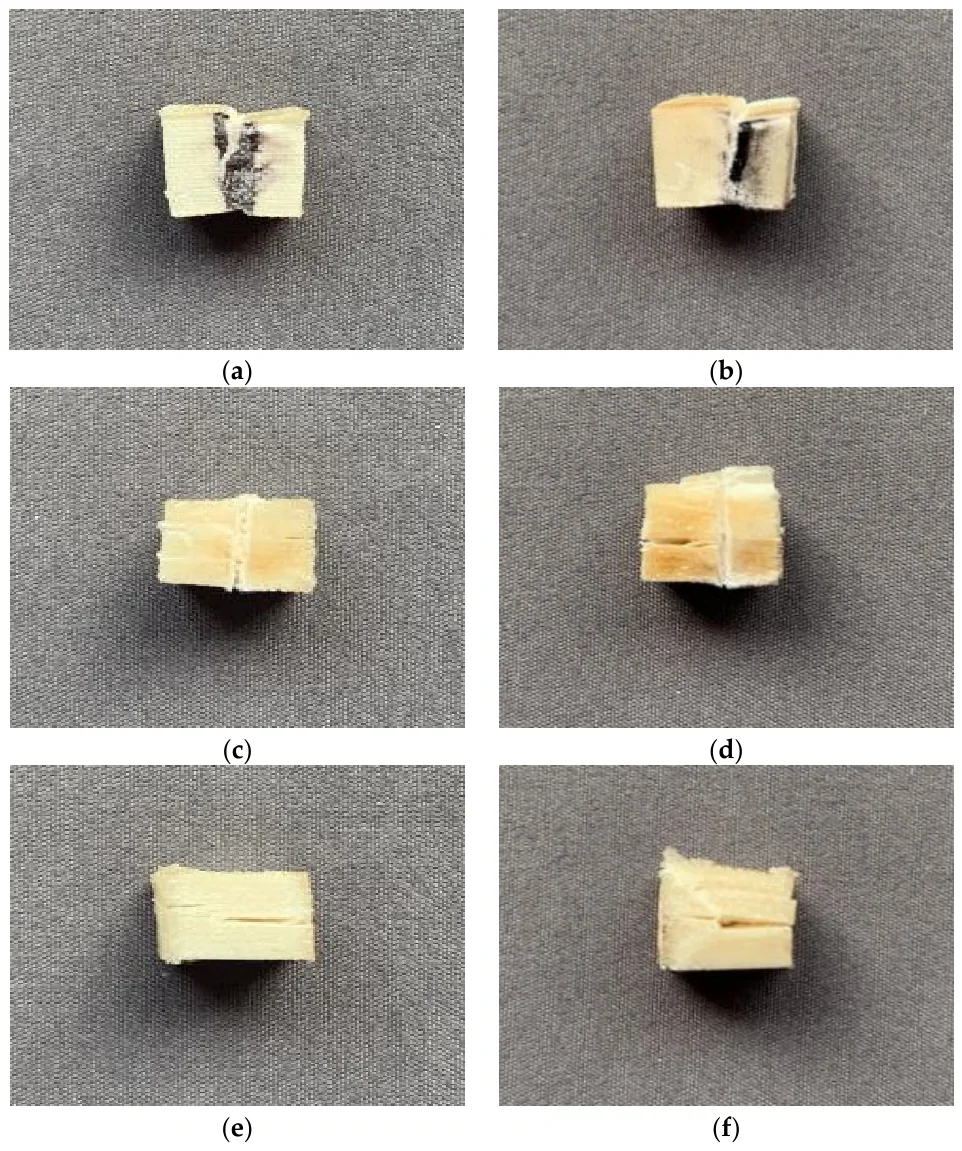
Section diagram of interlaminar shear breakage at 30 days of curing. (**a**) Front view of 40-ply laminate. (**b**) Front view of 50-ply laminate. (**c**) Top view of 40-ply laminate. (**d**) Top view of 50-ply laminate. (**e**) Side view of 40-ply laminate. (**f**) Side view of 50-ply laminate.

Figure 25 shows the cross-sectional views of interlaminar shear failure of specimens in layers 40 and 50 after 30 days of action. In addition to being affected by surface through cracks, a through crack appears in the compression zone of the 40-layer specimen, accompanied by other fine cracks. There is no compressive failure at the top of the compressed zone, indicating that the failure is greatly affected by stratification at this time. The surface cracks of the 50-layer specimens further expanded. Obvious cracks appeared at the top of the clamping end, accompanied by expansion. The top of the force-bearing zone expanded towards both ends due to compression damage, and the width of the vertical cracks further increased.

By comparing the specimens in Figure 20, Figure 21, Figure 22, Figure 23, Figure 24 and Figure 25, there is no obvious difference among the 10-layer specimens, all of which are compressive failures at the top of the compressed ends. The surface cracks of the 20-layer specimens after treatment were more obvious. Compared with the specimens that had not been treated, they had not yet developed to the compression zone and their surfaces were damaged by water molecules, resulting in the premature failure of the treated specimens. After the 30-layer specimens were subjected to action, the surface cracks of the specimens increased, and the extrusion damage at the top of the compression zone was obvious. The surface of the specimens was severely damaged after the action. After 30 days of action, the surface cracks of the 40-layer specimens developed severely, and multiple cracks appeared in the compression zone, indicating that water molecules had a marked impact on the interlayer damage of flax fiber cloth. The damage of the specimens changed from surface damage to damage caused by the combined action of surface and compression zone cracks. After the action, the surface cracks of the 50-layer plate increased. Due to the influence of water molecules, the clamping end, the surface of the specimen and the force-bearing area all cracked to varying degrees, which led to the failure of the specimen mainly caused by the development of cracks. After hand-laying 10 layers of boards, through cracks formed on the surface of the specimens that had not been treated, indicating that the surface of the treated specimens was affected by water molecules, reducing their influence.

Shear test failure begins with end compression failure. As the thickness of the specimen increases, the cracks in the compression zone further develop and their width also increases accordingly. When the number of fiber layers increases from 10 to 30, the failure of the specimens is caused by surface cracks. When it increases to 40 or 50 layers, the failure of the specimens is more affected by cracks in the compression zone.

With the extension of the aging time, the through cracks on the surface of the specimens gradually shift to the lower layers of the specimens. As the number of specimen layers increases, the failure of the specimens is not only affected by the through cracks on the surface, but also the cracking at the stressed end has a greater impact on the test results.

#### 3.3.3. Interlaminar Shear Test Results

**Figure 26:**
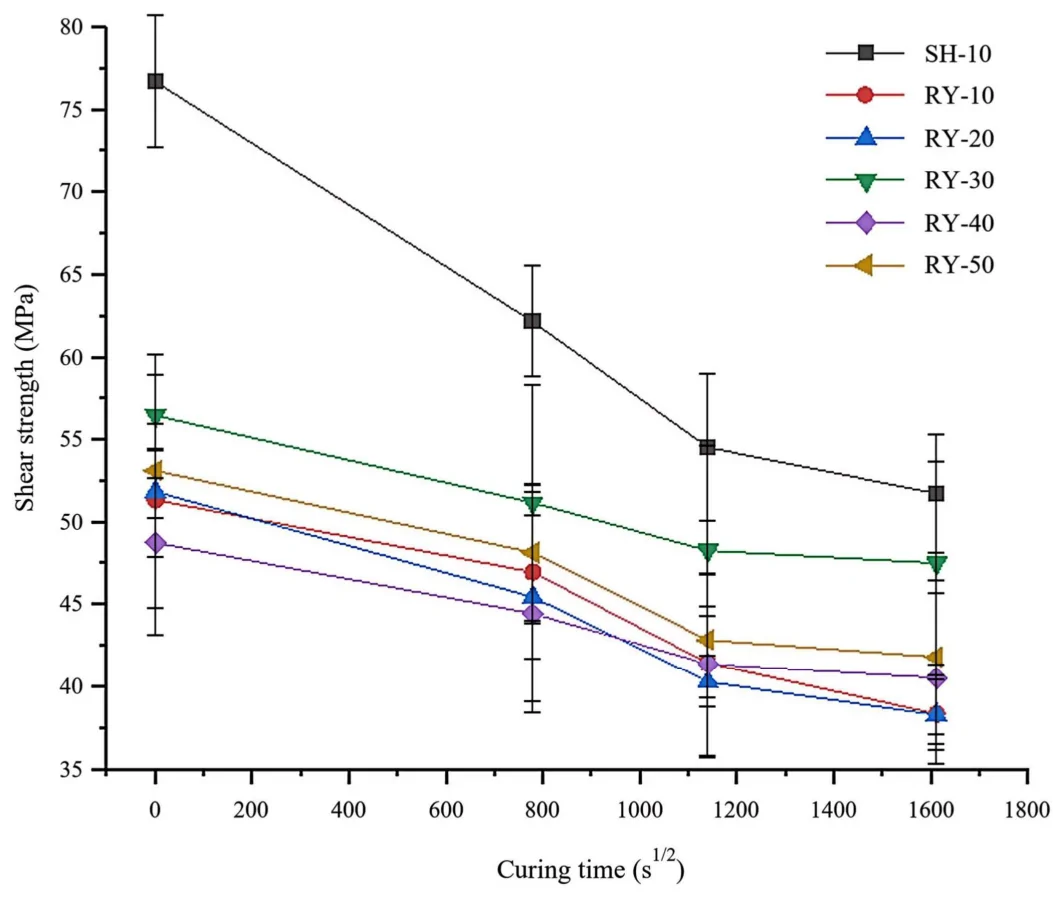
FFRP interlaminar shear strength test results.

Figure 26 shows the test results of interlaminar shear strength. The shear performance of NFRP made by the hand-laid molding process is higher than that of NFRP made by the autoclave molding process throughout the entire action cycle. However, the shear strength decreases the fastest during the action cycle, and the shear performance shows instability. The specimens made by the autoclave molding process have a lower shear strength than hand-laid boards, but they have better stability in a wet and hot environment, and the decrease in shear strength with the aging time is not marked. The interlaminar shear strength of the 30-layer specimens throughout the entire cycle of action was higher than that of the specimens of other layers, and the stability of the shear strength in the wet and hot environment was better than that of the specimens of other layers.

**Figure 27:**
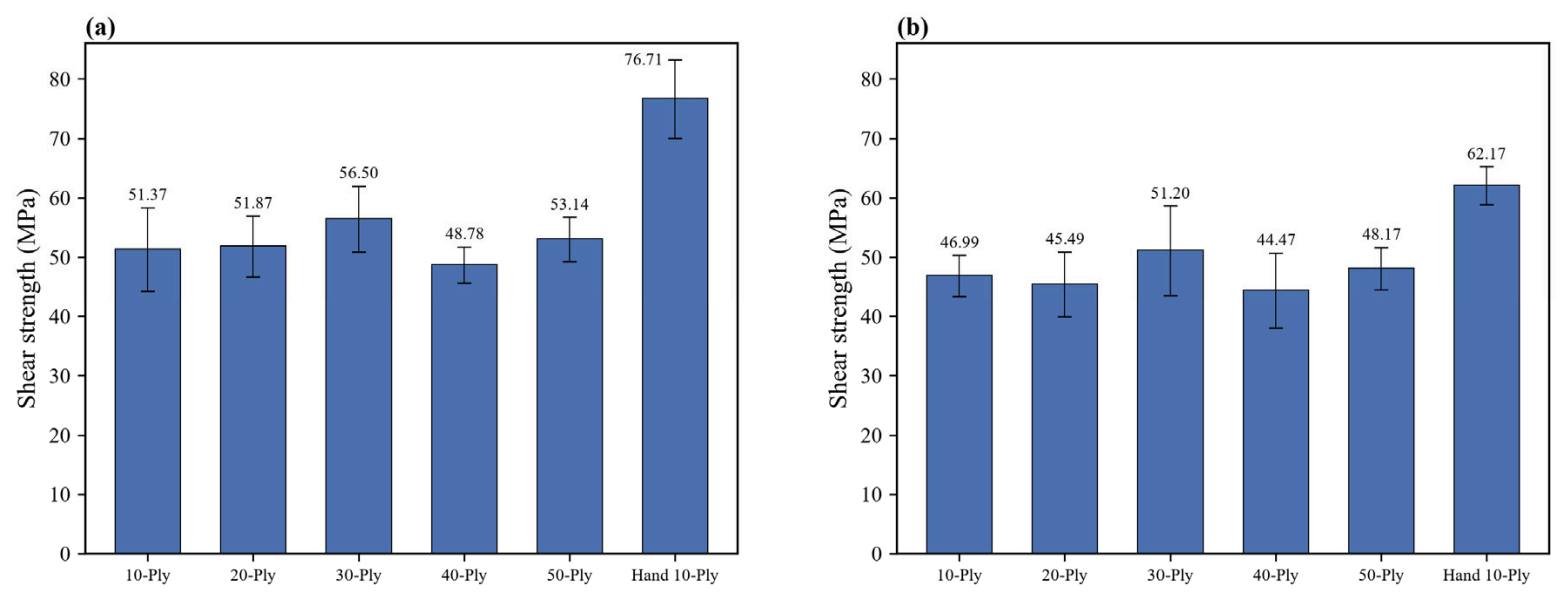

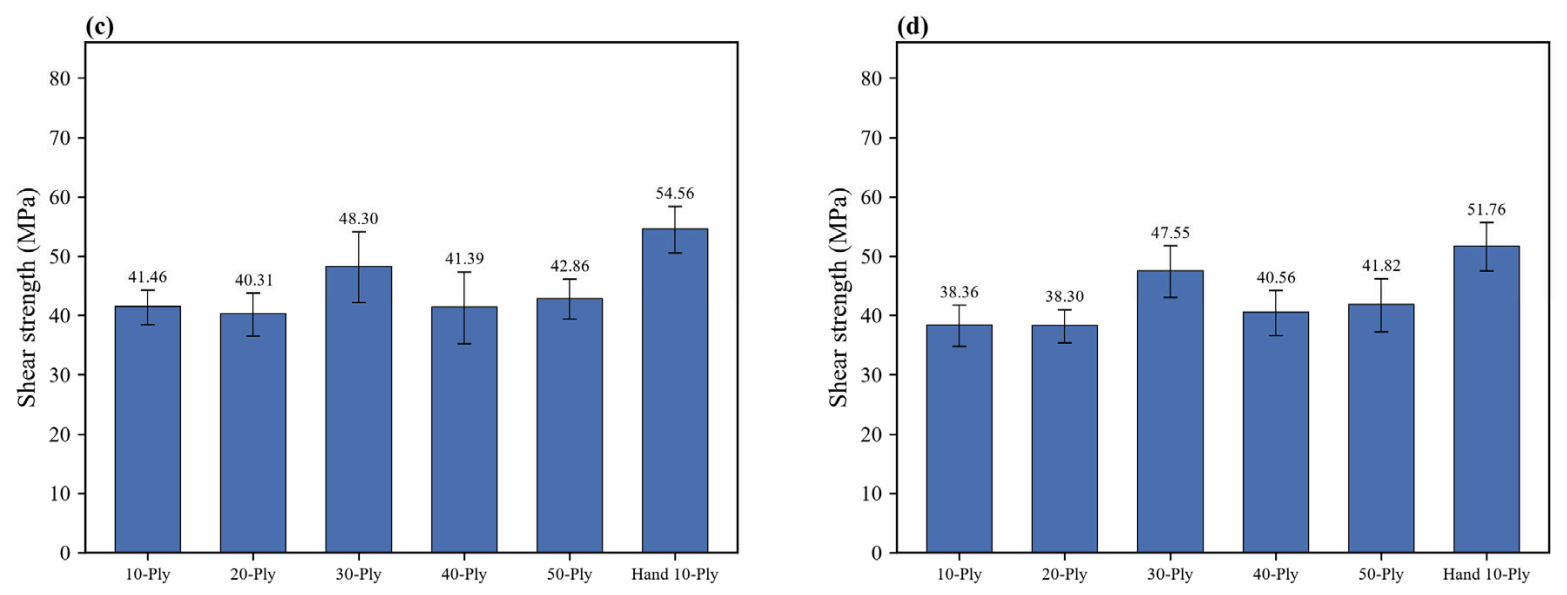
Comparison of internal shear strength under different curing environments. (**a**) Interlaminar shear strength at 0-day exposure. (**b**) Interlaminar shear strength at 7-day exposure. (**c**) Interlaminar shear strength at 15-day exposure. (**d**) Interlaminar shear strength at 30-day exposure.

Figure 27 shows the comparison of interlaminar shear strength under different operating conditions. Under 0d conditions, the interlaminar shear strength of the hand-laid molding process is 1.36 to 1.57 times that of the 10-layer autoclave process. At 7 days, the interlaminar shear strength of the hand-laid molding process FFRP is 1.21–1.40 times that of the autoclave, 1.13–1.35 times at 15 days, and 1.09–1.35 times at 30 days. The experimental phenomena show that the hand-laying process exhibits strong performance in the interlaminar shear test, but with the addition of working conditions, it shows a relatively large degradation rate. The degradation rate from 0 to 7 days was 18.90%, from 7 to 15 days was 12.24%, and from 15 to 30 days was 5.13%.

Compared with the hand-laying process FFRP, the degradation rate of the autoclave FFRP is lower throughout the entire application cycle, and its interlaminar shear performance is more stable in a wet and hot environment. Under the 0–7 d condition, the interlaminar shear degradation rate of the 10-layer FFRP is the lowest, at 8.5%. The degradation rate of the 20-layer FFRP is the highest, at 12.30%. Under the conditions of 7 d–15 d, 10-layer, 20-layer and 50-layer FFRP all showed relatively large degradation rates, which were 11.77%, 11.39% and 12.24% respectively. Under the conditions of 15 d–30 d, the interlaminar shear of FFRP in each layer number tends to be stable, and the degradation rate is not large.

Throughout the entire action cycle, the interlaminar shear strength of the 30-layer FFRP was higher than that of the other layers of FFRP. Moreover, the interlaminar shear strength degradation rate within each test time period was the lowest value, and the interlaminar shear strength degradation rate throughout the entire action cycle was 15.83%.

**Figure 28:**
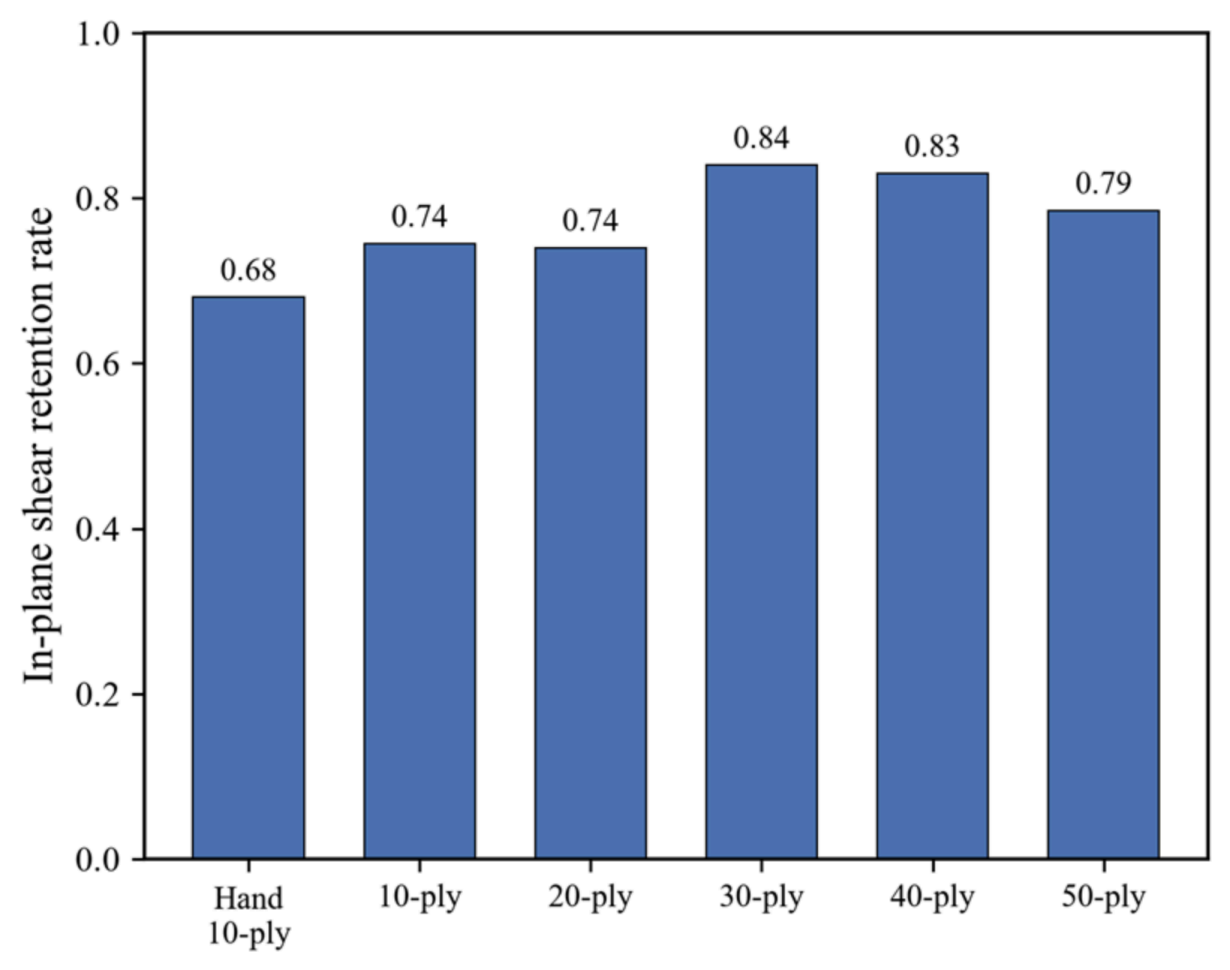
Interlayer shear retention rate of FFRP after 30 days of wet heat curing.

As shown in Figure 28, the interlaminar shear performance retention rate of the 10-layer FFRP board manufactured by the hand-laying process is the lowest. This is because the interlaminar defects caused by the manual laying of flax fiber cloth during the manufacturing process are greater than those of the FFRP board formed by the autoclave molding process. The retention rate of the FFRP plate formed by the 30-layer autoclave molding process is relatively high. The analysis suggests that the outer layer of fiber cloth may provide protection for the inner layer of fiber cloth, enabling the inner layer of fiber cloth to function well in the interlaminar shear test. With the increase in the number of fiber layers, the interlaminar shear performance shows a downward trend. It is believed that the aggregation of defects between fiber layers forms defect clusters. The impact of interlayer defects is greater than the contribution of outer fibers to the protection of inner fibers, and at this time, a negative mixing effect is manifested.

It should be noted that the interlaminar shear test was carried out under uniaxial compression with a custom fixture, rather than the short-beam three-point bending configuration of ASTM D2344, and the specimens were relatively thin, so the loaded end was prone to end-crushing and instability. Therefore, the above ‘interlaminar/interlaminar shear strength’ should be understood as a characteristic value under this specific compression-custom-fixture configuration, which is affected by multiple failure mechanisms including interlaminar shear, end-crushing and instability, and is not an absolute measurement of pure interlaminar shear strength. The interpretation of the shear strength results is therefore given as a relative comparison only, and is not cited quantitatively as the interlaminar shear strength under standard short-beam bending.

### 3.4. DMA Performance

In the preparation process of flax fiber composite materials, the formation of the interface between fibers and resins needs to go through two stages. The first stage is the wetting process of flax fibers and resin materials. Often, the wetting performance of the flax fiber surface will determine the strength of the interface performance of the composite material. The second stage is the curing process of the resin material, which achieves the solid state through the curing of molecular chain segments. Dynamic thermodynamic performance (DMA) tests can effectively measure the energy storage modulus (E′), damage modulus (E″), and loss factor (tan delta = E″/E′) of composite materials. Based on these parameters, the viscosity performance of flax fiber composite materials can be obtained.

The energy storage modulus, also known as the dynamic modulus, is related to the elastic modulus/Young’s modulus of the specimen. In practical processes, it represents the stiffness or brittleness of the material. This parameter serves as the potential or ability of the material to absorb energy during actual force application. The loss modulus, also known as the dynamic loss modulus, represents the viscous reaction of a material. It is a parameter that reflects the diffusion energy of the material in applications and is related to the internal friction of the material. The loss factor, as a dimensionless coefficient, represents the mechanical damping coefficient of the material.

The glass transition temperature of the specimen can be obtained through a DMA test. Generally, this involves the following factors: (1) the extreme temperature of the loss factor; (2) the temperature at the maximum damage modulus; (3) the temperature at the midpoint of the energy storage modulus–temperature curve; (4) if the energy storage modulus of the material increases with the increase in the test frequency under constant-temperature conditions, then this temperature is the glass transition temperature of the material. In this experiment, the extreme temperature of the loss factor is adopted as the glass transition temperature of FFRP.

Zhan Maosheng et al. found that the glass transition temperature (T) of CFRP decreased with the increase in test humidity in a wet and hot environment, and the higher the humidity, the more obvious the decrease in Tg. However, after the thermal oxidation aging test, the Tg ratio increased markedly. Paoanicolaoug et al. conducted research on the relationship between the mechanical and viscoelastic behavior of epoxy resin systems and the water absorption rate, temperature, and moisture absorption time. The research results show that T will markedly decrease with the passage of exposure time in a hot and humid environment and the increase in temperature. After drying the moisture-absorbing composite material, it was found that as the water molecules evaporated, the Tg ratio gradually increased, but the internal grid structure remained unchanged. Aying Zhangg analyzed and believed that in the initial stage of water absorption, due to the action of water molecules, the hydrogen bonds between molecules were broken, resulting in active chain segments at the same temperature and a decrease in the peak loss. Wang Zhanbin et al. found that with the extension of the duration of damp heat, the energy storage modulus showed a downward trend. The analysis suggested that it was related to the interface bonding, indicating a marked impact on mechanical properties.

#### 3.4.1. Test Parameters

The glass transition temperature of specimens with different layers was tested by using the dynamic thermomechanical analyzer model Q800 produced by TA Company (TA Instruments, New Castle, DE, USA). The heating rate was 5 °C/min, the vibration recombination frequency was 1 Hz, and the test temperature ranged from room temperature to 180 °C.

#### 3.4.2. DMA Test Curve

**Figure 29:**
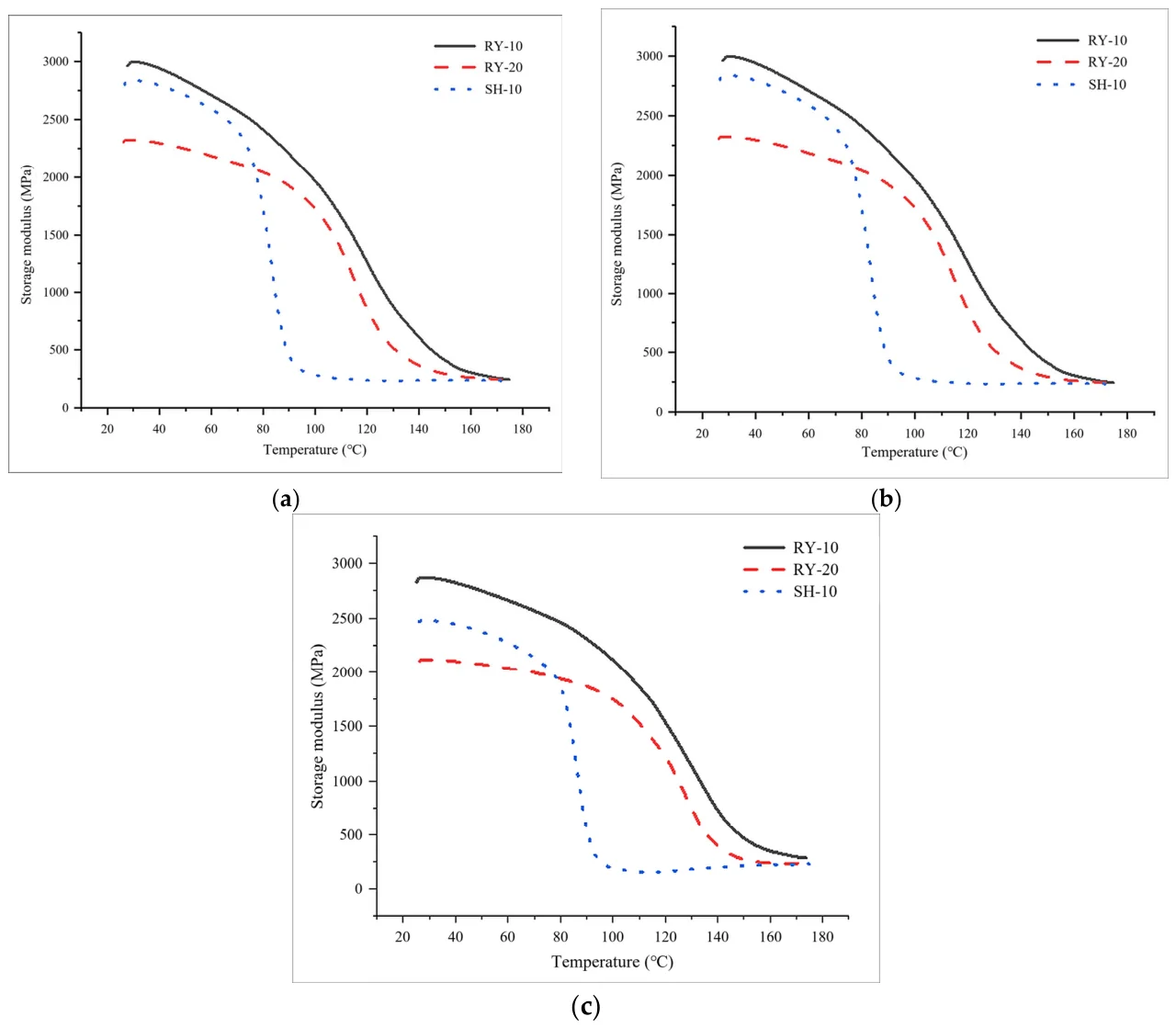
Energy storage modulus under different curing times. (**a**) Storage modulus at 0 days. (**b**) Storage modulus at 7 days. (**c**) Storage modulus at 15 days.

The DMA performance results of the FFRP composite material obtained through experiments in Section 2 are shown in Figure 29. As can be seen from the figure, with the extension of the aging time, the energy storage modulus of each test piece shows a downward trend. The reason for this phenomenon is that due to the diffusion effect of water molecules, the hydrogen bonds within the composite material break, increasing the freedom of the corresponding chain segments, and thus the energy storage modulus decreases. Under 0d conditions, the energy storage modulus of the 10-layer FFRP produced by the two molding processes is not markedly different, indicating that in the absence of action, the energy storage modulus is not greatly affected by the molding process. However, the energy storage modulus of the 20-layer FFRP is markedly lower than that of the 10-layer one. Under the condition of 7 days, the specimens in each group decreased compared with those in the 0-day environment. When 15 days are reached, the decrease in the energy storage modulus of FFRP formed by the hand-laying process is markedly faster than that of FFRP formed by the autoclave process. With the increase in the aging time, the energy storage modulus of each specimen showed a downward trend, indicating that the specimens were transforming from solid to liquid.

It should be noted that the glass transition temperature was determined from the peak (maximum) temperature of the loss factor (tan δ). As DMA characterization reflects a superposition of multiple failure and interfacial states, the results are used to compare the relative trends of different ply numbers and fabrication processes rather than as absolute quantitative values, and the corresponding conclusions are therefore relative comparisons.

**Figure 30:**
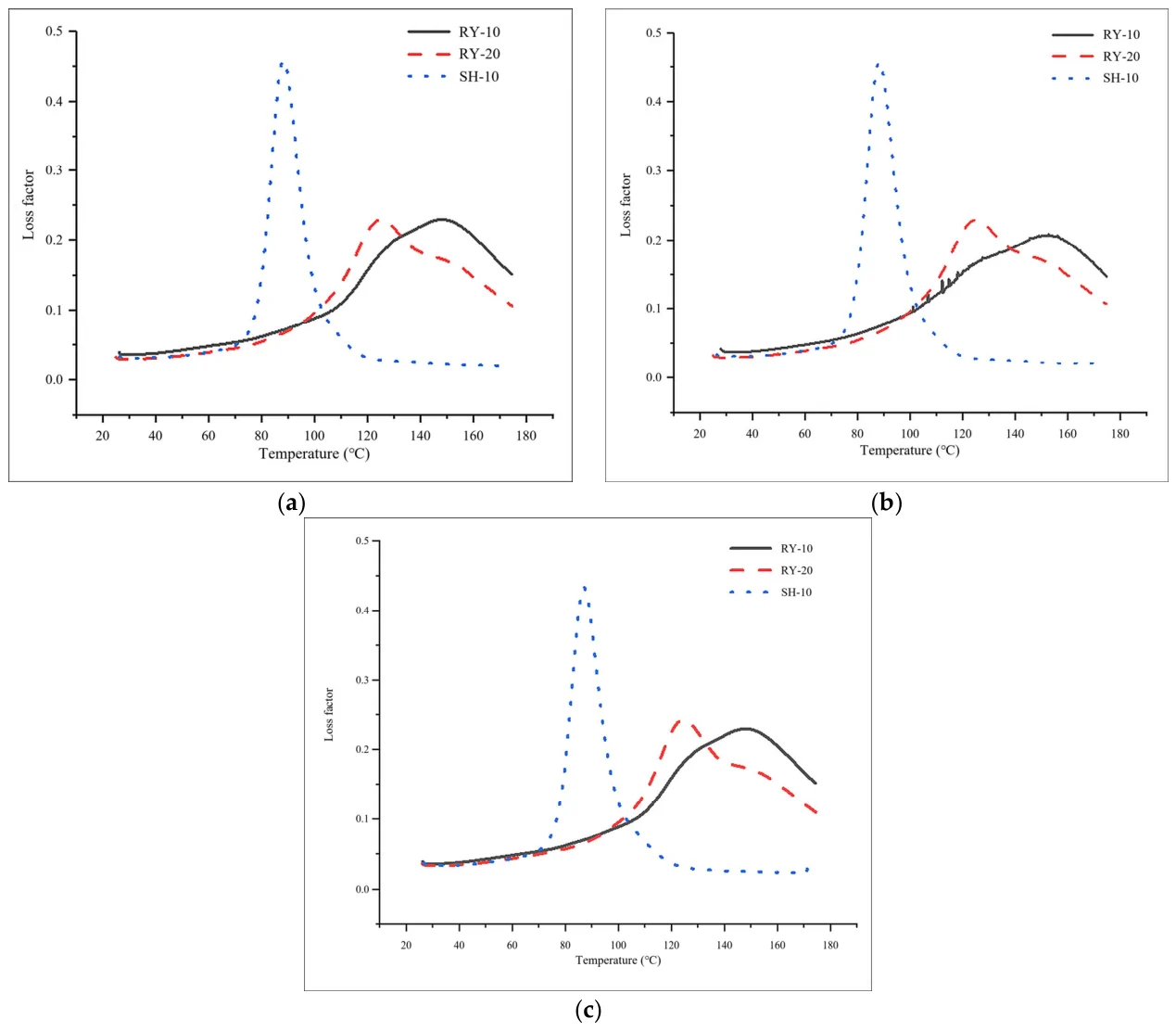
Loss factors under different maintenance times. (**a**) Loss factor at 0 days. (**b**) Loss factor at 7 days. (**c**) Loss factor at 15 days.

As can be seen from Figure 30a–c, with the increase in the aging time in the humid and hot environment, the extreme value of the loss factor shifts to the left, indicating that the temperature at the extreme value of the loss factor decreases. With the entry of water molecules, the resin matrix acts as a plasticizer, causing the matrix to swell, increasing the distance between molecules, breaking the van der Waals forces and hydrogen bonds between molecular chains, and reducing the intermolecular forces, resulting in a more obvious trend of intermolecular movement. It indicates that the flax fiber composite material after treatment can transform from a glassy state to a highly elastic state at a relatively low temperature. Further verification shows that as water molecules diffuse, Tg gradually decreases.

#### 3.4.3. Analysis of DMA Results

**Figure 31:**
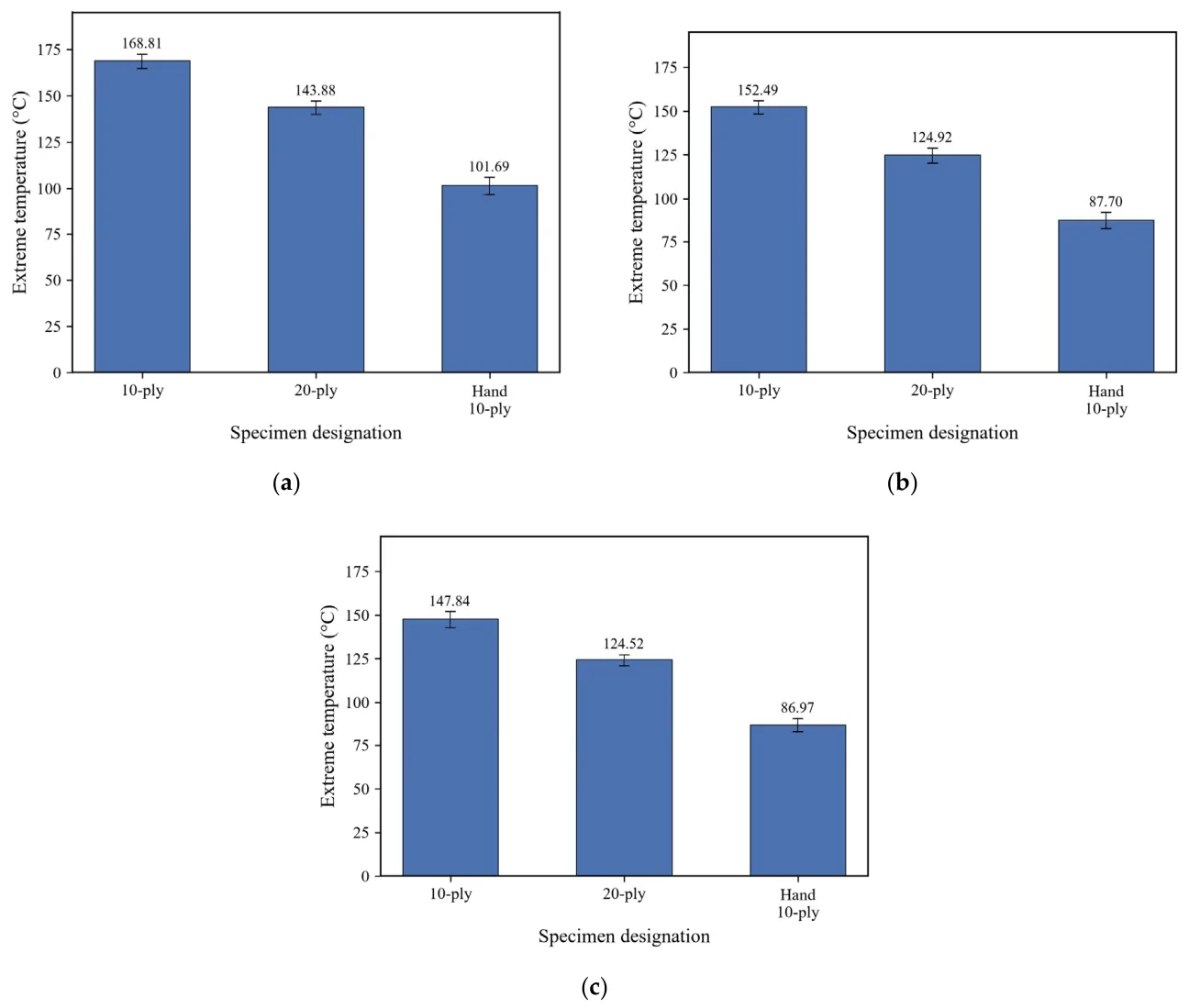
Extreme temperature of each specimen under different curing times. (**a**) Extreme temperature at 0 days. (**b**) Extreme temperature at 7 days. (**c**) Extreme temperature at 15 days.

Figure 31 shows the extreme temperature changes in each specimen at different aging times. It was found that with the increase in the aging time, the extreme temperatures of each specimen decreased. The extreme temperature drop of the 10-layer hot-pressed specimens after 7 days of action was 10.2%, that of the 20-layer specimens was 13.1%, and that of the 10-layer hand-laid specimens reached 14%. After 15 days of application, the reduction rate of the 10-layer hot-pressed specimens was 12.9%, that of the 20-layer hot-pressed specimens was 13.3%, and that of the 10-layer hand-laid specimens was 14.5%. It indicates that as the aging time increases, the rate of decline gradually decreases, and the rate is relatively large around 7 days of action. Under the same aging time, the glass transition temperature of the specimens made by the hand-laid molding process is lower than that of the specimens made by the autoclave molding process. In conclusion, it is believed that as water molecules diffuse, they cause frequent internal breakage of fibers and reduce intermolecular forces, thereby generating a larger free volume and transforming the composite material into a flexible state.

**Figure 32:**
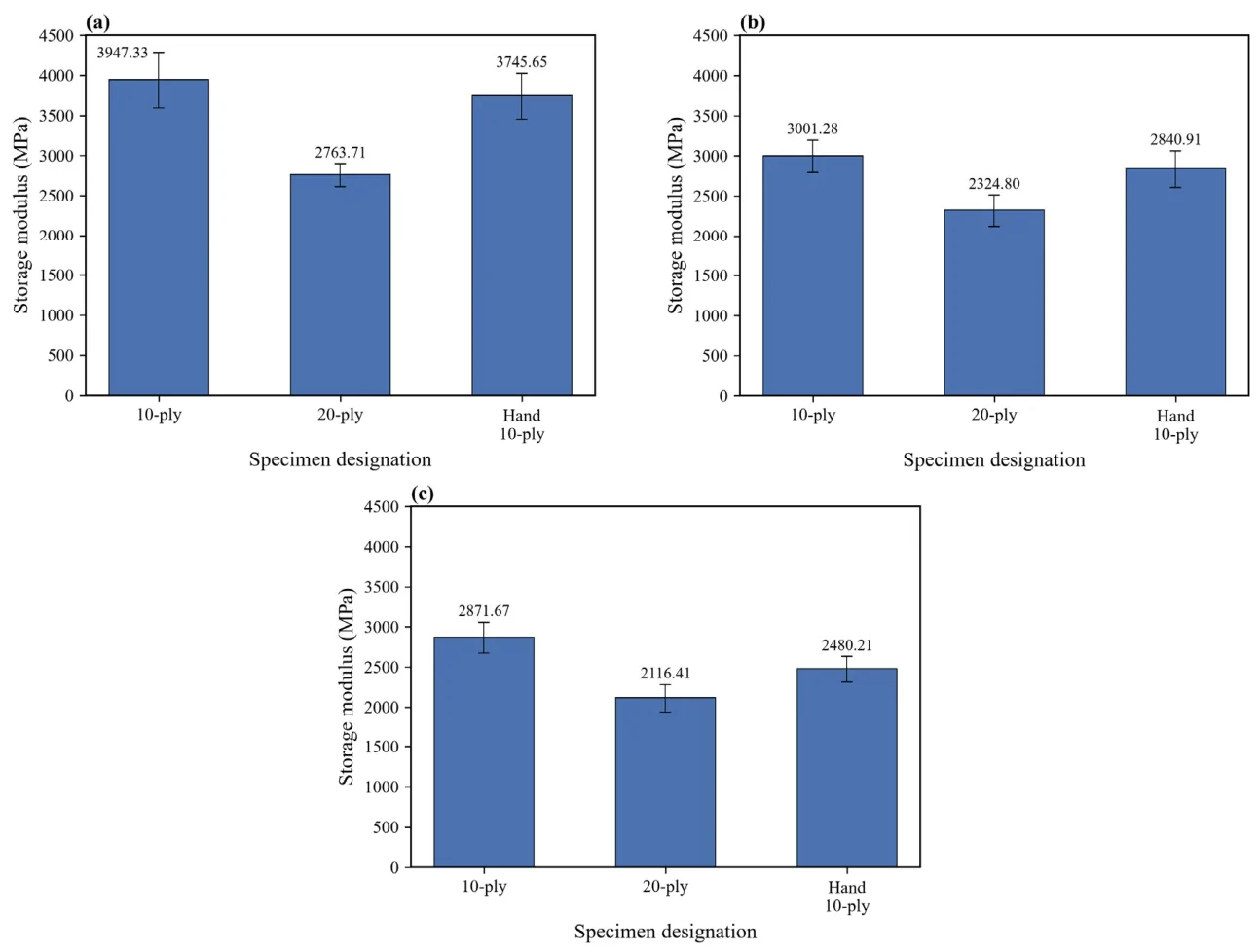
Energy storage modulus of each specimen under different curing times. (**a**) Storage modulus at 0 days. (**b**) Storage modulus at 7 days. (**c**) Storage modulus at 15 days.

**Figure 33:**
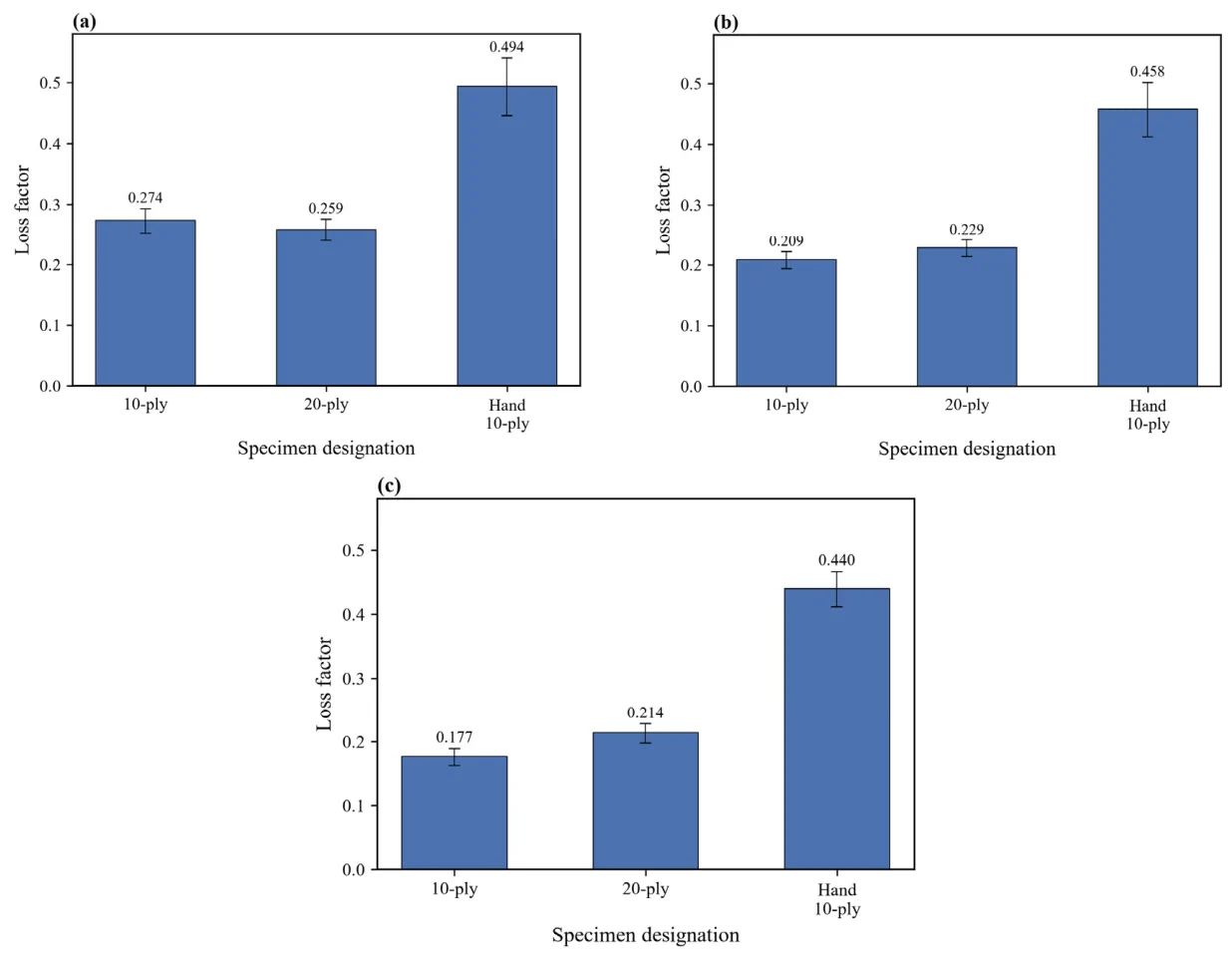
Loss factors of each specimen under different curing times. (**a**) Loss factor at 0 days. (**b**) Loss factor at 7 days. (**c**) Loss factor at 15 days.

As can be seen from Figure 32 and Figure 33, the temperatures at the extreme values of the energy storage modulus and loss factor in the 10th and 20th layers of the autoclave molding process and the 10th layer of the hand-laid FFRP decrease rapidly in the initial stage of wet heat action, and then tend to stabilize. Under the 0 d environment, the energy storage modulus of the two molding processes does not differ much because it is related to the interfacial performance of the composite material. The most crucial factor affecting the interfacial performance of the composite material is the effective connection between the interface caused by the parameters of the fibers and resins themselves, which is relatively less affected by the preparation molding process. With the increase in application time, the water absorption rate of FFRP formed by the hand-laying process is higher than that of 10-layer FFRP formed by the autoclave process due to it possessing more internal pores. Therefore, the declining trend in its energy storage modulus is faster than that of the autoclave molding process.

As the exposure time to a damp and hot environment increases, the resin matrix cracks, the fibers and resin de-bond, and the interface cracks. The free water absorbed by water is converted into bound water. The combined factors lead to a decrease in the overall Tg after the damp and hot effect. By comparing the two sets of test materials of the autoclave molding process, it was found that the energy storage modulus and glass transition temperature of the 20-layer FFRP were both lower than those of the 10-layer FFRP under the same aging time. The analysis suggests that under the same forming process, as the number of fiber layers increases, the internal pores of the fibers accumulate, making it easier for water molecules to combine with hydrogen bonds on the fiber surface under the action of a wet and hot environment.

### 3.5. SEM Test Results

**Figure 34:**
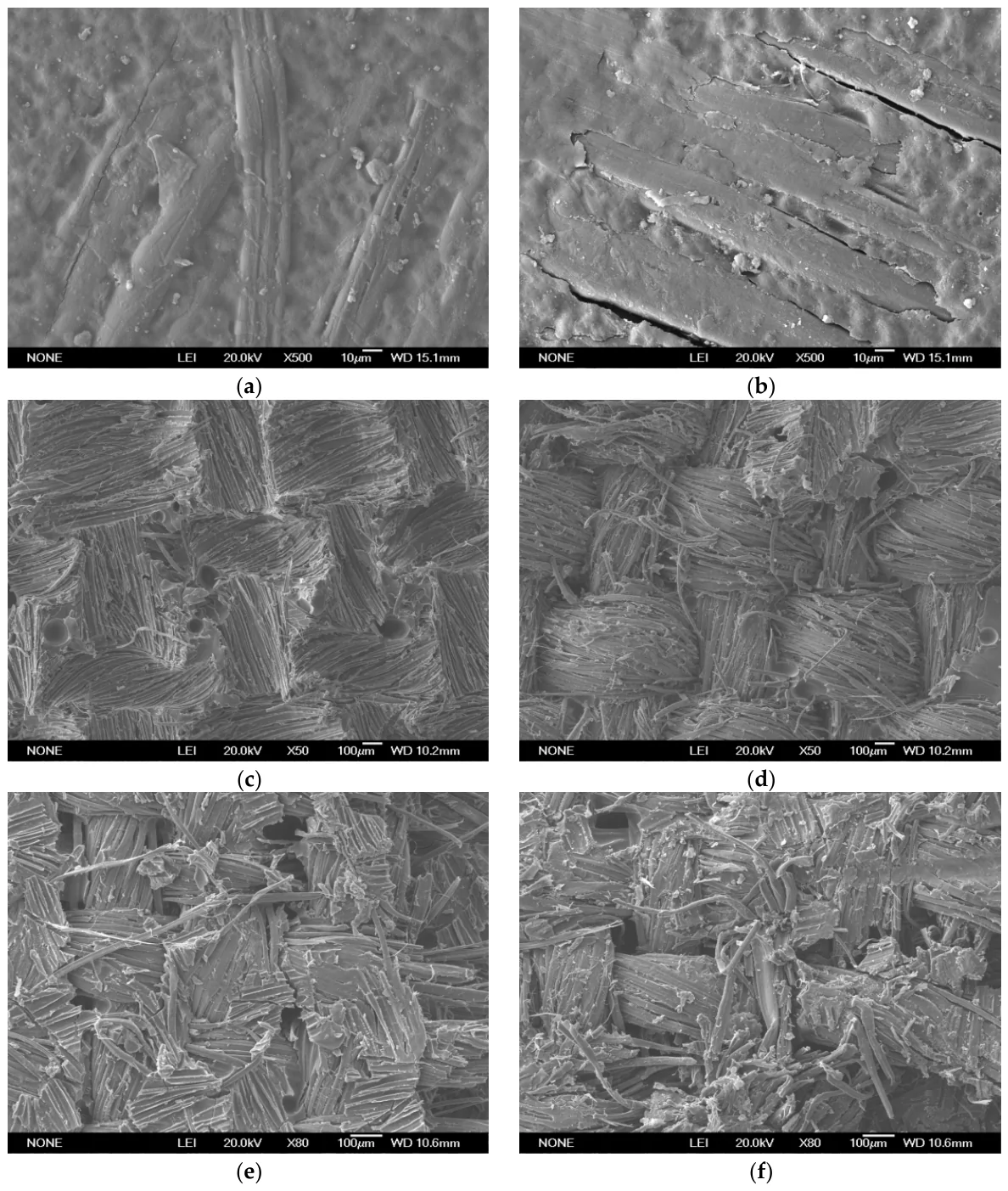
Scanning electron microscopy results of each test under different curing times. (**a**) Non-hygrothermal conditioned water absorption specimen. (**b**) Thirty-day hygrothermal conditioned water absorption specimen. (**c**) Non-hygrothermal conditioned interlaminar shear specimen. (**d**) Thirty-day hygrothermal conditioned interlaminar shear specimen. (**e**) Non-hygrothermal conditioned tensile specimen. (**f**) Thirty-day hygrothermal conditioned tensile specimen.

This section mainly focuses on the scanning electron microscopy analysis of the water absorption test, interlaminar shear test and tensile test before and after the test, respectively analyzing the surface changes in the specimens without treatment and after 30 days of treatment. The results of the scanning electron microscope are shown in Figure 34.

It is found from Figure 34a that the fibers on the surface of the uncured specimen are well combined with the resin, with few surface cracks and no obvious scattered impurities on the surface. The few cracks observed at this time are mainly caused by manufacturing errors. In Figure 34b, it is observed that the number of cracks on the surface of the specimen has markedly increased, and the width of the cracks has further expanded. Moreover, there is a phenomenon of resin and fiber detachment, and the detached resin is in block form.

Figure 34c,d are the specimen characterization diagrams after the interlaminar shear test without action and after 30 days of action, respectively. From Figure 34c, it is found that the yarns are relatively tight and there are fewer burrs on the shear failure surface, indicating that the failure mainly results from yarn breakage and interlayer failure. It was observed in Figure 34d that the burrs on the shear failure surface increased, and the yarn spacing also increased. The yarn arrangement in the shear test failure cross-section was relatively sparse. It indicates that the shear failure of the specimens after action is mainly caused by the failure of the interface between the yarn and the resin.

**Figure 35:**
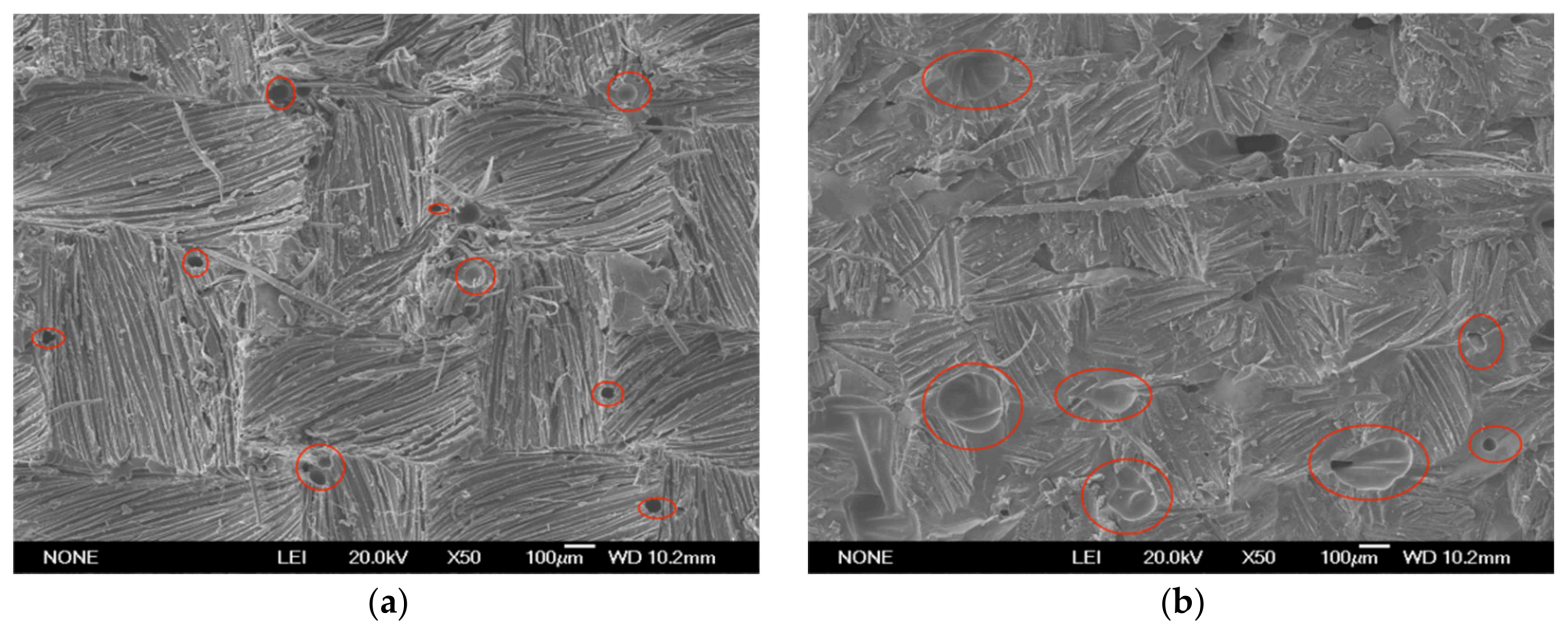
Comparison of internal defects of different specimens: (**a**) 10-ply specimen; (**b**) 50-ply specimen.

Figure 34e,f respectively show the tensile test characterization diagrams without action and after 30 days of action. Observation revealed that the surface fibers of the specimens that were not subjected to tensile testing broke more frequently after the test. The arrangement direction of the fibers after breaking was relatively scattered, and the spacing of the fiber bundles on the surface of the specimens was more affected by the tensile test compared to before the tensile test. Figure 34f shows the tensile test failure characterization under a 30-day environment. It is observed that the surface failure of the specimen is mostly fiber pull-out, and the distribution of fiber fracture directions is relatively concentrated. By comparing the specimens that were not subjected to action, it was observed that the interlayer connection of the fibers in the specimens was good when they were not subjected to action, and no fiber pull-out phenomenon occurred. Most of them were fiber fractures. After the test piece was subjected to action, the interface between the fibers and the resin was damaged due to the entry of water molecules, showing signs of fiber extraction. Figure 35 presents the scanning electron microscopy results of the 10-layer and 50-layer specimens in the unopened state.

From Figure 35a, it is found that 10 layers of specimens are randomly distributed with tiny holes. This is because during the specimen forming process, the internal air was not completely expelled. It was found from Figure 35b of 28 that the holes in the 50-layer specimens were larger and more concentrated. This was due to the fact that during the manufacturing process, as the thickness of the fiber layers increased, the compressive strength of the internal layers of the fibers was relatively low, and the internal air could not be expelled. As a result, the internal air accumulated and formed large bubbles, which in turn affected its mechanical properties. After the scanning electron microscopy analysis of the water absorption, and interlaminar shear and tensile tests before and after the above-mentioned tests, it was found that the interface between the fibers and the resin of the specimens after the tests was damaged due to water molecules, which led to the fact that the damage of each test was greatly affected by the fiber pull-out. Moreover, as the aging time increases, the spacing of the fiber bundles in both the radial and weft directions increases to varying degrees, which is also one of the reasons for the decline in mechanical properties.

## 4. Conclusions

In a humid and hot environment, the mechanical properties of flax fiber composites are unstable. Taking different fiber layers and different molding processes as variables, water absorption, interlaminar shear, tensile and DMA tests were conducted on the sheet performance of FFRP. The degradation laws of the mechanical properties of FFRP with different layers in a humid and hot environment at 40 °C and 90% relative humidity were analyzed. The degradation of the mechanical properties of FFRP with different numbers of layers is summarized as follows.

Analysis of the water absorption rate of the plates shows that the water absorption rates of the 30-layer and 20-layer FFRP water absorption specimens are consistent in the later stage. The water absorption rate of the 20-layer specimens tends to be saturated, reaching a measured overall (total) water uptake of about 4.5% at 30 days, while the 30-ply FFRP still showed an upward trend. It is noted that this measured total uptake is markedly higher than the fitted Fickian saturation value M_m reported in the fitting parameters (Table 6), which is consistent with the two-stage (Fickian + relaxation) water absorption behavior discussed in Section 3.1.3. It should be noted that, because the specimens differ considerably in thickness and therefore in diffusion path length, specimens of different thicknesses do not reach an equivalent moisture state within the same aging duration; hence, the lower apparent water absorption of the thicker 30-layer specimen at 30 days partly reflects slower diffusion rather than a purely superior durability.

The influence of damp heat conditions on the tensile properties of FFRP sheets is mainly reflected in that the tensile strength degradation rate and tensile modulus reduction value of FFRP composite materials formed by the 30-layer autoclave molding process are both lower than those of FFRP sheets of other layers, indicating that under the conditions of 40 °C temperature and 90% relative humidity, the tensile performance of 30-layer sheets is better.

The main influence of wet heat conditions on the interlaminar shear performance of FFRP boards is that after the wet heat test of 30 and 40 layers of FFRP, the retention rate of interlaminar shear strength both reached over 80%, indicating that the interlaminar shear strength of FFRP with this number of layers is least affected by the wet heat environment.

The DMA test results of the FFRP sheet after the damp heat test show that with the increase in the damp heat effect time, both the energy storage modulus and the glass transition temperature show a downward trend. For FFRP with the same molding process but different layers, the decreasing trends in the energy storage modulus and glass transition temperature are positively correlated with water absorption rate. Electron microscopy (SEM) analysis also suggested that the interface between the fibers and the resin of the specimens after the action was damaged by water molecules. With the increase in the aging time, the fiber bundle spacing in both the radial and weft directions increased to varying degrees.

## Figures and Tables

**Table 1:** Density and mechanical properties of plant fibers and synthetic fibers.

Fiber	Density (g/cm^3^)	Tensile Strength (MPa)	Tensile Modulus (GPa)	Specific Strength (MPa/g·cm^3^)	Specific Modulus (GPa/g·cm^3^)	Elongation at Break (%)
Ramie fiber	1.5	400–938	44–128	270–620	29–85	2.0–3.8
Flax fiber	1.5	345–1830	27–80	230–1220	15–53	1.2–3.2
Hemp fiber	1.5	550–1110	58–70	370–740	39–47	1.6
Jute fiber	1.3–1.5	393–800	10–55	300–610	7.1–39	1.5–1.8
Sisal fiber	1.3–1.5	507–855	9.4–28	362–610	6.7–20	2.0–2.5
Cotton–flax blended fiber	1.5–1.6	287–800	5.5–13	190–530	3.7–8.4	3.0–10
Coconut coir fiber	1.2	131–220	4–6	110–180	3.3–5	15–30
E-glass fiber	2.55	4000–5000	70	800–1400	29	2.5
Carbon fiber	1.75	2000–4000	250–500	1710	164–171	1.4–1.8

**Table 2:** Properties of epoxy resin used to prepare NFRP for hand-laid plates.

	Viscosity (MPa·s)	Color	Curing Time
Solution A	11,000–13,000	Colorless or pale yellow	—
Solution B (Hardener)	11	Colorless	—
A:B (1:0.345)	600–700	Pale yellow	3–6 h

**Table 3:** Process parameters of autoclave prepreg.

Project	Indicator
Surface density of prepregs	233 ± 20 (g/m^2^)
Fiber areal density	140 ± 10 (g/m^2^)
Volatile matter content	<2 (%)
Resin content	40 ± 3 (%)

**Table 4:** Comparison of the two fabrication processes.

Item	Hand-Laying	Autoclave Molding
Resin system	TS-impregnating adhesive (tensile strength 65.65 MPa, modulus 3.19 GPa)	AGMP3600 bio-based resin (rosin anhydride-based)
Fiber form	Bidirectional flax fabric (Harbin Changli)	RP140 flax fabric (prepreg)
Resin content (%)	60 ± 5%	40 ± 4%
Fiber areal density	140 ± 10 g/m^2^	140 ± 10 g/m^2^
Molding pressure	0.1 MPa	0.5–0.6 MPa
Curing	Room-temperature curing	130 °C, 120 min
Ply numbers	10 layers (control)	10/20/30/40/50 layers
Relative thickness	About 2.4 times the autoclave-molded sheet	Thinner

**Table 5:** Test matrix: number of independent specimens for each test condition, defined as one combination of manufacturing method, ply number and aging period. * For the water absorption test, the same set of specimens was weighed repeatedly at all aging periods; the numbers marked with an asterisk are the independent specimens per (manufacturing method × ply number) group, not per aging period.

Test	Manufacturing Method	Ply Number	Aging Period	Specimens per Test Condition	Total
Water absorption	Autoclave	10/20/30/40/50	0, 12 h, 24 h, 3 d, 15 d, 30 d	11 *	55
Water absorption	Hand-laying	10	0, 12 h, 24 h, 3 d, 15 d, 30 d	12 *	12
Tensile	Autoclave	10/20/30/40/50	0/7/15/30 d	3	60
Tensile	Hand-laying	10	0/7/15/30 d	3	12
Interlaminar shear	Autoclave	10/20/30/40/50	0/7/15/30 d	5	100
Interlaminar shear	Hand-laying	10	0/7/15/30 d	5	20
DMA	Autoclave	10/20	0/7/15 d	10	60
DMA	Hand-laying	10	0/7/15 d	8	24

**Table 6:** Water absorption fitting parameters of FFRP at 90% relative humidity.

Specimen	M_m_ (%)	D (×10^−5^ mm^2^/s)
Hand-laid 10-ply	0.71	1.11
Autoclave-molded 10-ply	0.52	2.12
Autoclave-molded 20-ply	1.01	2.36
Autoclave-molded 30-ply	0.80	1.39
Autoclave-molded 40-ply	1.05	1.99
Autoclave-molded 50-ply	1.85	2.00

The fitted M_m values in Table 6 are lower than the final water content measured experimentally (e.g., the 20-ply specimen reached about 4.5%). This is consistent with the two-stage behavior of the water-uptake curves in Figure 13: Equation (1) is a single-term (single-Fickian) fit, so M_m_ captures only the asymptotic saturation of the Fickian diffusion component and does not include the non-Fickian water uptake induced by the later matrix/interface relaxation. Mm and D are therefore used as relative indicators of the diffusion rate and hygroscopic tendency, rather than as the true equilibrium moisture content.

## Data Availability

The original contributions presented in this study are included in the article. Further inquiries can be directed to the corresponding author.
